# LRK-1/LRRK2 and AP-3 regulate trafficking of synaptic vesicle precursors through active zone protein SYD-2/Liprin-α

**DOI:** 10.1371/journal.pgen.1011253

**Published:** 2024-05-09

**Authors:** Sravanthi S. P. Nadiminti, Shirley B. Dixit, Neena Ratnakaran, Anushka Deb, Sneha Hegde, Sri Padma Priya Boyanapalli, Sierra Swords, Barth D. Grant, Sandhya P. Koushika

**Affiliations:** 1 Department of Biological Sciences, Tata Institute of Fundamental Research, Mumbai, Maharashtra, India; 2 Department of Molecular Biology and Biochemistry, Rutgers University, Piscataway, New Jersey, United States of America; Brown University, UNITED STATES

## Abstract

Synaptic vesicle proteins (SVps) are transported by the motor UNC-104/KIF1A. We show that SVps travel in heterogeneous carriers in *C*. *elegans* neuronal processes, with some SVp carriers co-transporting lysosomal proteins (SV-lysosomes). LRK-1/LRRK2 and the clathrin adaptor protein complex AP-3 play a critical role in the sorting of SVps and lysosomal proteins away from each other at the SV-lysosomal intermediate trafficking compartment. Both SVp carriers lacking lysosomal proteins and SV-lysosomes are dependent on the motor UNC-104/KIF1A for their transport. In *lrk-1* mutants, both SVp carriers and SV-lysosomes can travel in axons in the absence of UNC-104, suggesting that LRK-1 plays an important role to enable UNC-104 dependent transport of synaptic vesicle proteins. Additionally, LRK-1 acts upstream of the AP-3 complex and regulates its membrane localization. In the absence of the AP-3 complex, the SV-lysosomes become more dependent on the UNC-104-SYD-2/Liprin-α complex for their transport. Therefore, SYD-2 acts to link upstream trafficking events with the transport of SVps likely through its interaction with the motor UNC-104. We further show that the mistrafficking of SVps into the dendrite in *lrk-1* and *apb-3* mutants depends on SYD-2, likely by regulating the recruitment of the AP-1/UNC-101. SYD-2 acts in concert with AP complexes to ensure polarized trafficking & transport of SVps.

## Author summary

Synaptic vesicles (SVs) at neuronal synapses have a defined membrane composition and size. We show that SV proteins (SVps) are transported in carriers with heterogenous composition, with a small subset containing lysosomal proteins (SV-lysosomes). We propose that the SV-lysosomes are perhaps post-Golgi trafficking intermediates from which the SV proteins are sorted away from lysosomal proteins through the action of LRK-1, the ortholog of LRRK2, which is associated with both familial and sporadic Parkinson’s disease, and the AP-3 complex, which is associated with Hermansky-Pudlak syndrome. These sorting events could be necessary events for the formation of SV precursors.

SVp carriers and SV-lysosomes both depend on the UNC-104/KIF1A kinesin motor and the active zone protein SYD-2/Liprin-α for their transport. However, in the absence of the AP-3 complex, altered trafficking of SV and lysosomal proteins likely causes the SV-lysosomes to become more dependent on the UNC-104-SYD-2 complex for their transport, while the SVp carriers lacking lysosomal proteins become partially independent of UNC-104. We therefore propose that SYD-2 can act as a link between the upstream trafficking events and the subsequent transport of SVps likely through its interaction with the motor UNC-104. We also show that SYD-2 genetically interacts with and regulates the localization of the AP-1 complex to facilitate polarised distribution of SVp carriers to axons. Thus, our study highlights novel roles for SYD-2 in several early steps of SVp trafficking.

## Introduction

Synaptic vesicles (SVs) found at the pre-synaptic terminal contain membrane-associated proteins, such as Synaptobrevin-1 (SNB-1), Synaptogyrin-1 (SNG-1), SV2, and RAB-3 [1]. They are known to have a well-defined composition lacking, for instance, Golgi-resident enzymes [1–3]. The loss of SV proteins (SVps) has been shown to affect neurotransmission [4–8] and the progression of neurodegenerative disorders [9]. However, the trafficking routes of SVps in the cell body remain to be fully elucidated. Although SNB-1 and SNG-1 are present along with RAB-3 at synapses, only a subset of the SNB-1 and SNG-1 carriers that exit the cell body include RAB-3 [3,10]. Likewise, Synaptophysin and SV2 do not appear to be co-transported by the mammalian SV motor KIF1A [11], while Synaptophysin and the Zinc transporter ZnT3 are likely enriched in different populations of synaptic-like microvesicles [2]. Additionally, SVp carriers exiting the cell body are tubular as opposed to those closer to the synapse, which have a smaller diameter [12, 13]. Prior studies from mammalian cells and *Drosophila* suggest that some SVps share trafficking routes with lysosomal proteins [14–16]. These findings suggest that SVps emerge from the cell body in precursor or immature transport carriers that likely have a heterogeneous composition, with some sharing trafficking routes with lysosomal proteins.

Several genes have been identified as important in the trafficking of SVps. UNC-16/JIP3-mediated recruitment of LRK-1/LRRK2 on the Golgi seems to be critical for excluding Golgi-resident enzymes from SVp carriers as well as regulating the size of these carriers [3]. The AP-3 complex has been shown to play a key role in separating SVps and lysosomal proteins that initially occupy a common intermediate compartment [14]. The biogenesis and maturation of precursor vesicles containing the endolysosomal protein LAMP-1, active zone proteins, and SV proteins are regulated by RAB-2 [17]. UNC-104/KIF1A is the kinesin motor important for SVp transport [11,18–20]. We have previously shown that the SVp carriers formed in the *unc-16*/*jip3*, *lrk-1*/*lrrk2*, and *apb-3* (mutant of the β subunit of the AP-3 complex) mutants of *Caenorhabditis elegans* are not exclusively dependent on UNC-104/KIF1A for their transport [3]. However, the link between the maturation of SVp carriers and their ability to recruit the SVp motor remains to be well understood.

Active zone proteins that mark release sites for SVs at synapses have also been shown to co-transport with some SVps [15,21–23]. Moreover, SVps and some active zone proteins, such as ELKS-1, have been shown to co-transport in lysosomal protein-containing packets called pre-synaptic lysosome-related vesicles (PLVs). These PLVs are dependent on the small GTPase ARL-8, an interactor of UNC-104/KIF1A/IMAC, which is thought to facilitate UNC-104/KIF1A’s association with PLVs [15]. Additionally, active zone proteins Piccolo and Bassoon present in clusters with Synaptobrevin, Synaptotagmin, and SV2, are thought to be important in forming such transport clusters [24]. Together, these data suggest that SVp and lysosomal protein trafficking and transport can be regulated by active zone proteins.

SYD-2/Liprin-α, an active zone protein [25,26], is known to interact with and bind to the SV motor UNC-104/KIF1A [27–30]. SYD-2/Liprin-α also influences the distribution of acidic organelles such as SVs [27], dense core vesicles [31], and lysosomes [32]. Active zone proteins SYD-2 and SYD-1 along with synapse assembly proteins SAD-1 and CDK-5 are known to regulate lysosomal protein trafficking in *unc-16* mutants through dynein [32]. ELKS-1, which binds SYD-2 [33,34], has been shown to interact with RAB-6 to regulate the trafficking of melanosomal proteins [35] and SVs [36]. These studies suggest that SYD-2 can affect the trafficking of SVs and other acidic organelles.

In this study, we used the *C*. *elegans* touch receptor neuron (TRN) model to better define the co-transport and eventual separation of SV and lysosomal proteins. Importantly, we show that LRK-1 and the AP-3 complex, which we previously identified as important for regulating SV precursor composition [3], play a critical role in sorting lysosomal proteins and SVps. Furthermore, the active zone protein SYD-2/Liprin-α plays a key role along with UNC-104/KIF1A in the transport of compartments containing both SVps and lysosomal proteins in the absence of the AP-3 complex. Our data suggest that although the SV motor can be recruited on compartments that contain both SVps and lysosomal proteins (SV-lysosomes), SV precursors lacking lysosomal proteins appear to preferentially recruit the SV motor UNC-104.

## Results

### Synaptic vesicle proteins travel with lysosomal proteins in heterogenous carriers

Although studies have indicated that SVps are transported in heterogeneous carriers, the composition of these carriers has not been fully examined. Here, we assessed the co-transport of specific SVps with one another and with other endomembrane compartment proteins in the proximal posterior lateral mechanosensory (PLM) neuron of *C*. *elegans* (S1A–S1G and S2A Figs and S1 and S2 Movies). As markers for the endomembrane compartments, we used MAN-II, a Golgi resident enzyme, to label the Golgi complex [37]; the lysosomal cysteine transporter CTNS-1 [38], and the endolysosomal markers RAB-7 and LMP-1 [39,40].

**Fig 1:**
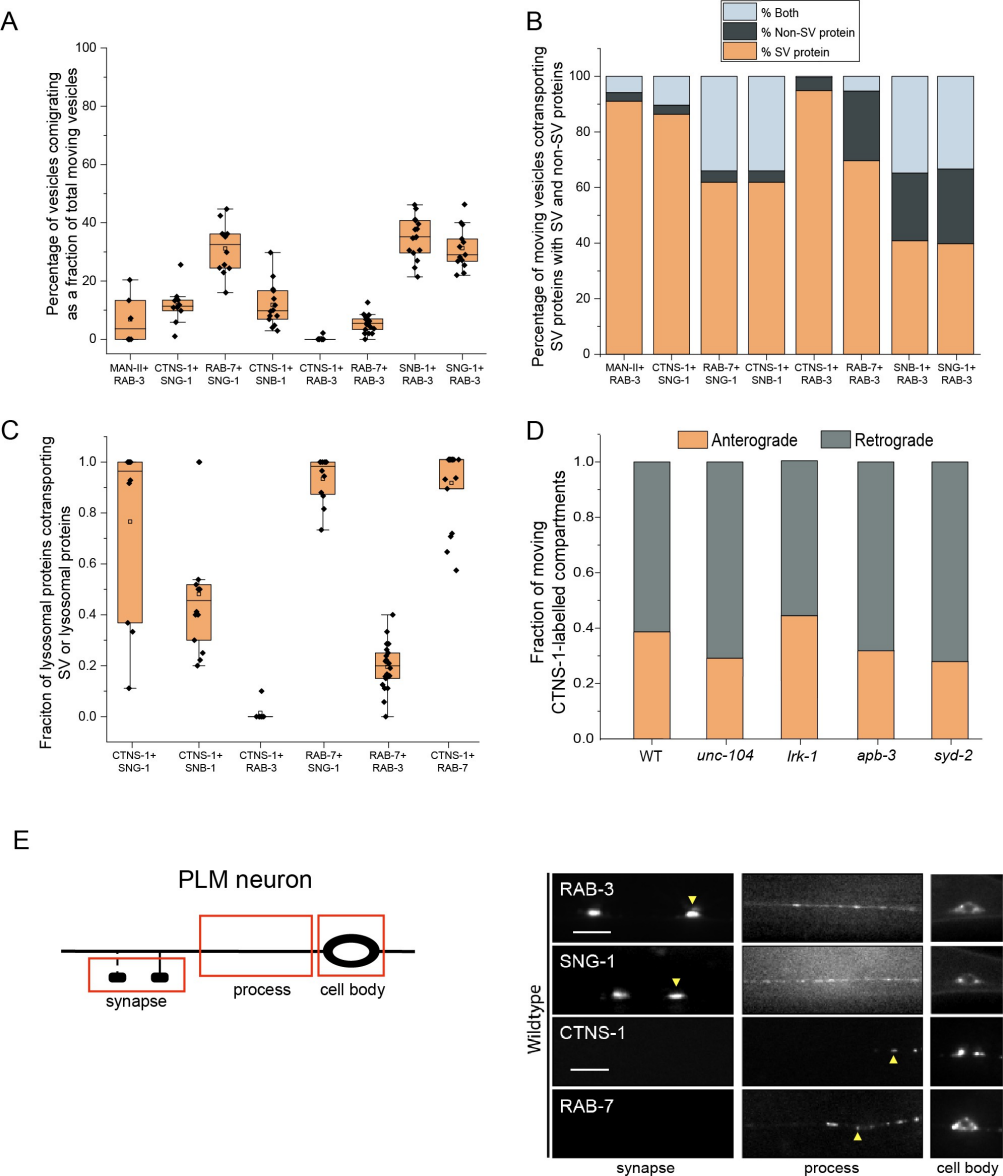
Synaptic vesicle proteins travel with lysosomal proteins in heterogenous carriers. (A) Quantitation of percentage of total moving vesicles co-transporting different combinations of synaptic vesicle proteins and lysosomal proteins from kymograph analysis of dual color imaging. The number of animals per genotype (N) ≥ 10; number of vesicles analyzed (n) > 600. (B) Quantitation of percentage of total moving vesicles transporting different synaptic vesicle proteins or lysosomal proteins or both from kymograph analysis of dual color imaging. The number of animals per genotype (N) ≥ 10; number of vesicles analyzed (n) > 600. (C) Quantitation of fraction of various total moving lysosomal proteins co-transporting different synaptic vesicle proteins from kymograph analysis of dual color imaging. N ≥ 10; n > 100. (D) Quantitation of fraction of total moving CTNS-1-labeled compartments moving in the anterograde and retrograde direction in different mutants. N ≥ 9 per genotype; the number of CTNS-1-labeled compartments ≥ 20. (E) Schematic of the PLM neuron. Red boxes indicate the regions of imaging. The arrow shows the anterograde direction of vesicle motion. (F) GFP::RAB-3, SNG-1::GFP, CTNS-1::mCherry, and RAB-7::mScarlet in the cell body, process, and synapses of wildtype PLM neurons. Scale bar: 10 μm.

SVp carriers in the neuronal process largely exclude Golgi-resident enzymes, endolysosomal & lysosomal proteins (Fig 1A and 1B). However, there is a small proportion of SVp carriers that co-transports lysosomal proteins, while most of the lysosomal protein carrying compartments in the neuronal process co-transport SV proteins (Fig 1C). We will hereafter refer to the SVp carriers that cotransport lysosomal proteins as SV-lysosomes. Since nearly all CTNS-1-labelled compartments co-transport SNG-1 (Fig 1C), we consider the CTNS-1-marked compartments as the SV-lysosomes. The SV-lysosomes predominantly move in the retrograde direction (Fig 1D) and are largely restricted to the cell body and the axonal region proximal to the cell body (Fig 1E). Interestingly, only the transmembrane SVps co-transport as SV-lysosomes, as RAB-3 is almost completely excluded from compartments transporting CTNS-1, suggesting that RAB-3 perhaps marks a subset of SVp-only carriers (Fig 1A–1C). Majority of the CTNS-1 compartments, from animals expressing CTNS-1 alone or in combination with other transgenes, show similar range of velocity in both anterograde and retrograde directions (S2B Fig). Compartments transporting only CTNS-1 in a strain expressing both CTNS-1 and SNG-1 move with velocities comparable to compartments co-transporting SNG-1 and CTNS-1 (S2C Fig). CTNS-1 compartments that do not co-transport SNG-1 are often smaller than those co-transporting both markers (S2D Fig).

In wildtype animals, both CTNS-1 and RAB-7 labelled SV-lysosomes are localized to the first 25 μm of the neuronal process and never reach the PLM synapse (Figs 1E, S1H and S1I).

Thus, SVps travel in heterogeneous transport carriers, with a small number going with lysosomal proteins, in carriers that we refer to as the SV-lysosomes, that do not travel far into the neuronal process. We think that the SV-lysosomes represent a post-Golgi trafficking intermediate from which SVps and lysosomal proteins are separately trafficked to pre-SVs and lysosomes by LRK-1 and the AP-3 complex.

### LRK-1 and the AP-3 complex exclude lysosomal proteins from SVp transport carriers

Mammalian and *Ce* orthologs of LRK-1 and the AP-3 complex have been shown to regulate trafficking of SV and lysosomal proteins [14,36–38,3]. Therefore, we investigated whether these genes regulate the trafficking of the SV-lysosomes in *C*. *elegans* TRNs.

**Fig 2:**
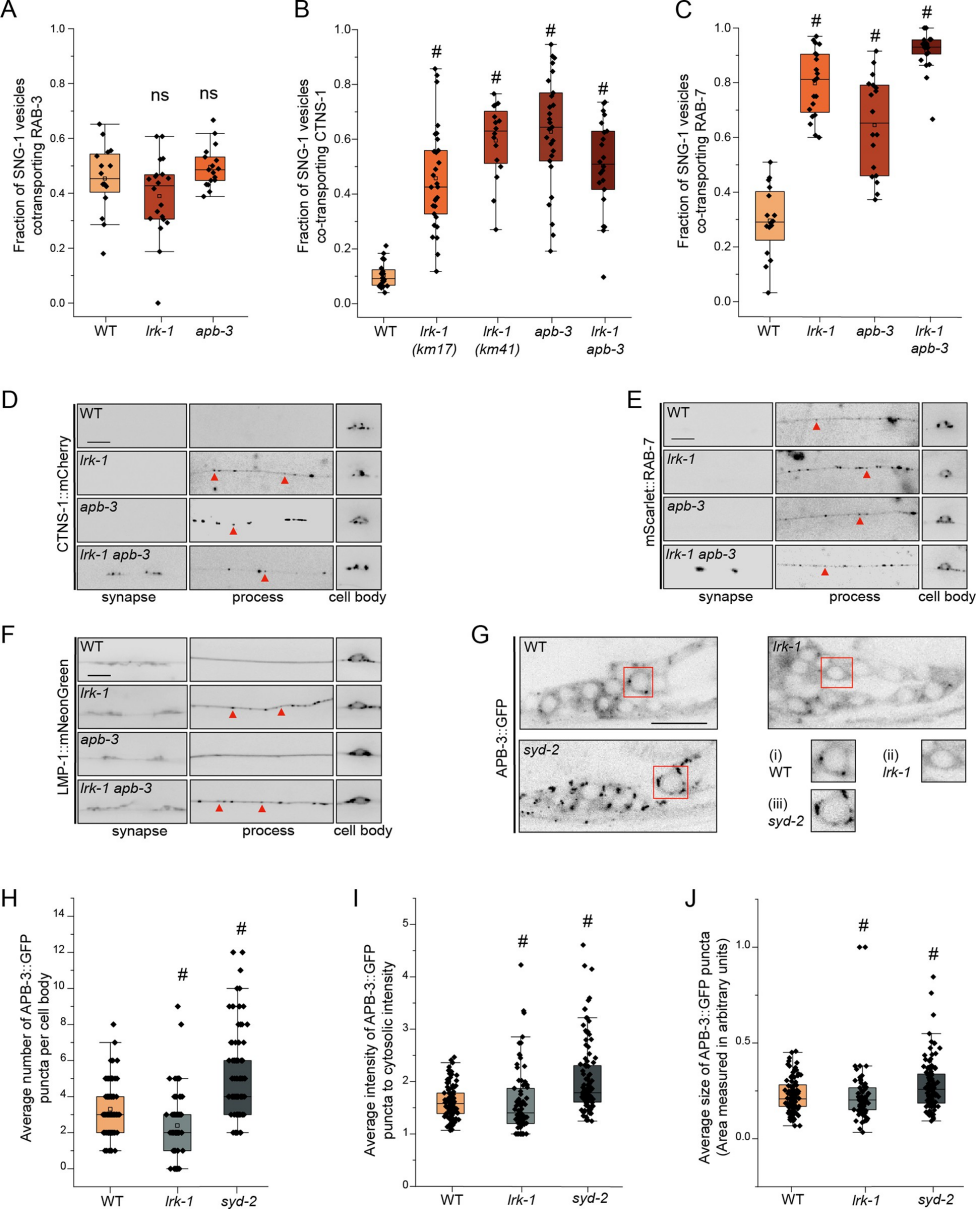
LRK-1 and AP-3 act in parallel and through SYD-2 to regulate lysosomal protein trafficking. (A) Quantitation of fraction of total moving SNG-1-carrying vesicles co-transporting RAB-3 in WT, *lrk-1*(*km17*), and *apb-3*(*ok429*) from kymograph analysis of sequential dual color imaging at 1.3 frames per second (fps). # P-value ≤ 0.05 **(**One-Way ANOVA with Tukey’s post-hoc test, all comparisons to WT); ns: not significant; Number of animals (N) ≥ 15 per genotype; number of vesicles analyzed per genotype (n) > 800. (B) Quantitation of fraction of total moving SNG-1-carrying vesicles co-transporting CTNS-1 in WT, *lrk-1*(*km17*), *lrk-1*(*km41*), *apb-3*(*ok429*) and *lrk-1*(*km17*) *apb-3*(*ok429*), from kymograph analysis of sequential dual color imaging at 1.3 fps. # P-value ≤ 0.05 (Mann–Whitney Test); N ≥ 15 per genotype; n > 500. (C) Quantitation of fraction of total moving SNG-1-carrying vesicles co-transporting RAB-7 from WT, *lrk-1*(*km17*), *apb-3*(*ok429*) and *lrk-1*(*km17*) *apb-3*(*ok429*), kymograph analysis of dual color imaging. # P-value ≤ 0.05 **(**One-Way ANOVA with Tukey’s post-hoc test, all comparisons to WT); ns: not significant; N ≥ 20 per genotype; n > 800. (D) CTNS-1::mCherry in the cell body, process, and synapses of PLM neurons of WT, *lrk-1*(*km17*), *apb-3*(*ok429*), and *lrk-1*(*km17*) *apb-3*(*ok429*). Scale bar: 10 μm. Red arrows point to some CTNS-1-labeled compartments. (E) mScarlet::RAB-7 in the cell body, process, and synapses of PLM neurons of WT, *lrk-1*(*km17*), *apb-3*(*ok429*), and *lrk-1*(*km17*) *apb-3*(*ok429*). Scale bar: 10 μm. Red arrows point to some RAB-7-labeled compartments. (F) LMP-1::mNeonGreen in the cell body, process, and synapses of PLM neurons of WT, *lrk-1*(*km17*), *apb-3*(*ok429*), and *lrk-1*(*km17*) *apb-3*(*ok429*). Scale bar: 10 μm. Red arrows point to some RAB-7-labeled compartments. (G) Images show APB-3::GFP puncta in the head ganglion cell bodies of WT, *lrk-1*(*km17*), and *syd-2*(*ok217*). Scale bar: 10 μm. Red boxes highlight the regions of insets with cell bodies from images show APB-3::GFP in (i) WT, (ii) *lrk-1*, and (iii) *syd-2*. (H) Quantitation of the number of APB-3::GFP puncta per cell body in WT, *lrk-1*(*km17*), and *syd-2*(*ok217*). # P-value ≤ 0.05 (Mann–Whitney Test); ns: not significant; N > 10 animals; n > 75 cell bodies. (I) Quantitation of intensity of APB-3::GFP puncta in cell bodies of WT, *lrk-1*(*km17*), and *syd-2*(*ok217*). The ratio of the intensity of APB-3::GFP puncta to cytosolic intensity in the cell body is plotted. # P-value ≤ 0.05 (Mann–Whitney Test); ns: not significant; N > 10 animals; n > 75 cell bodies. (J) Quantitation of average size of APB-3::GFP puncta per cell body in WT, *lrk-1*(*km17*), and *syd-2*(*ok217*). # P-value ≤ 0.05 (Mann–Whitney Test); ns: not significant; N > 10 animals; n > 75 cell bodies.

We’ve previously assessed the SVp carrier membrane composition by measuring the co-transport of two SVps SNB-1 and RAB-3 [3]. Mutants of *lrk-1* and *apb-3* show reduced incidence of co-transport of SNB-1 and RAB-3, suggesting that the trafficking of SNB-1 and RAB-3 to a common SVp carrier is regulated by LRK-1 and AP-3 [3]. However, the co-transport of SNG-1 and RAB-3 remains largely unaffected in *lrk-1* and *apb-3* mutants as compared to wildtype animals (Fig 2A).

There is a significant increase in the co-transport of the SVp SNG-1 with CTNS-1 and RAB-7 in *lrk-1* and *apb-3* mutants as compared to wildtype (Figs 2B and 2C, and S3A–S3F, and S4–S6 Movies), suggesting that more SV-lysosomes are transported out of the cell body in these mutant animals. Notably, the co-transport of CTNS-1 with SNB-1 is not affected in *lrk-1* and *apb-3* mutants (S4A Fig), and RAB-3 continues to be absent from CTNS-1-carriers in these mutant animals (S4B and S4C Fig), suggesting that LRK-1 and the AP-3 complex play key roles in trafficking of SNG-1-containing SV-lysosomes, and that RAB-3 may continue to mark the SVp-only carriers in these mutants. In *lrk-1* and *apb-3* mutants, more SV-lysosomes localize farther in the neuronal process than in wildtype (Figs 2D, 2E, S1H and S1I). SV-lysosomes travel farther out of the cell body of *lrk-1 apb-3* double mutants than they do in either single mutant of *lrk-1* and *apb-3* (Figs 2D, 2E, S1H and S1I). Further, trafficking of another endolysosomal protein, LMP-1, is only affected in *lrk-1* mutants (Figs 2F and S1J) suggesting that LRK-1 may act upstream of APB-3 in the trafficking of SV-lysosomes. Interestingly, CTNS-1-positive SV-lysosomes are longer in *apb-3* mutants than in WT or *lrk-1* mutants (S4D Fig), suggesting that the AP-3 complex also affects the size of SV-lysosomes.

Thus, LRK-1 and AP-3 regulate sorting of lysosomal proteins and some SVps at the SV-lysosome sorting intermediate. In the absence of these genes, the SV-lysosomes persist and travel along the neuronal process likely dependent on their ability to recruit more motors for their transport.

### LRK-1 regulates localization of the AP-3 complex

LRK-1 acts via the AP-1 and the AP-3 complexes to regulate polarized SVp trafficking and the trafficking of SVp transport carriers [3,41]. LRK-1 is known to assist in the Golgi membrane localization of the AP-1 clathrin adaptor complex, thereby regulating its function [3,42,43]. To examine whether LRK-1 regulates the membrane localization of the AP-3 complex as well, we examined the distribution of the β subunit of the AP-3 complex, APB-3::GFP, in neuronal cell bodies of *lrk-1* mutants (Fig 2G). In wildtype, APB-3::GFP shows punctate localization in the cell body. APB-3::GFP puncta in *lrk-1* mutants are fewer, while their intensity and size is similar to that in WT (Figs 2G–2J and S4E). This suggests that in *lrk-1* mutants the AP-3 complex may not be recruited efficiently to membrane surfaces. Some of the sorting roles of LRK-1 are likely mediated by facilitating AP-3 localization to membrane surfaces.

### SV-lysosomes in *lrk-1* and *apb-3* mutants are differentially dependent on UNC-104

SVps are known to be dependent on the anterograde motor UNC-104/KIF1A for their exit from neuronal cell bodies [11,18,20,44]. In *Drosophila* neurons, SV-lysosomes depend on ARL-8, a known facilitator of IMAC/KIF1A-mediated transport [15]. The transport of SVp carriers in *lrk-1* and *apb-3* mutants is only partially dependent on UNC-104 [3]. Therefore, we characterized the role of UNC-104 in transporting both SVs and SV-lysosomes out of neuronal cell bodies using a cargo binding-defective alleles of *unc-104* [44].

**Fig 3:**
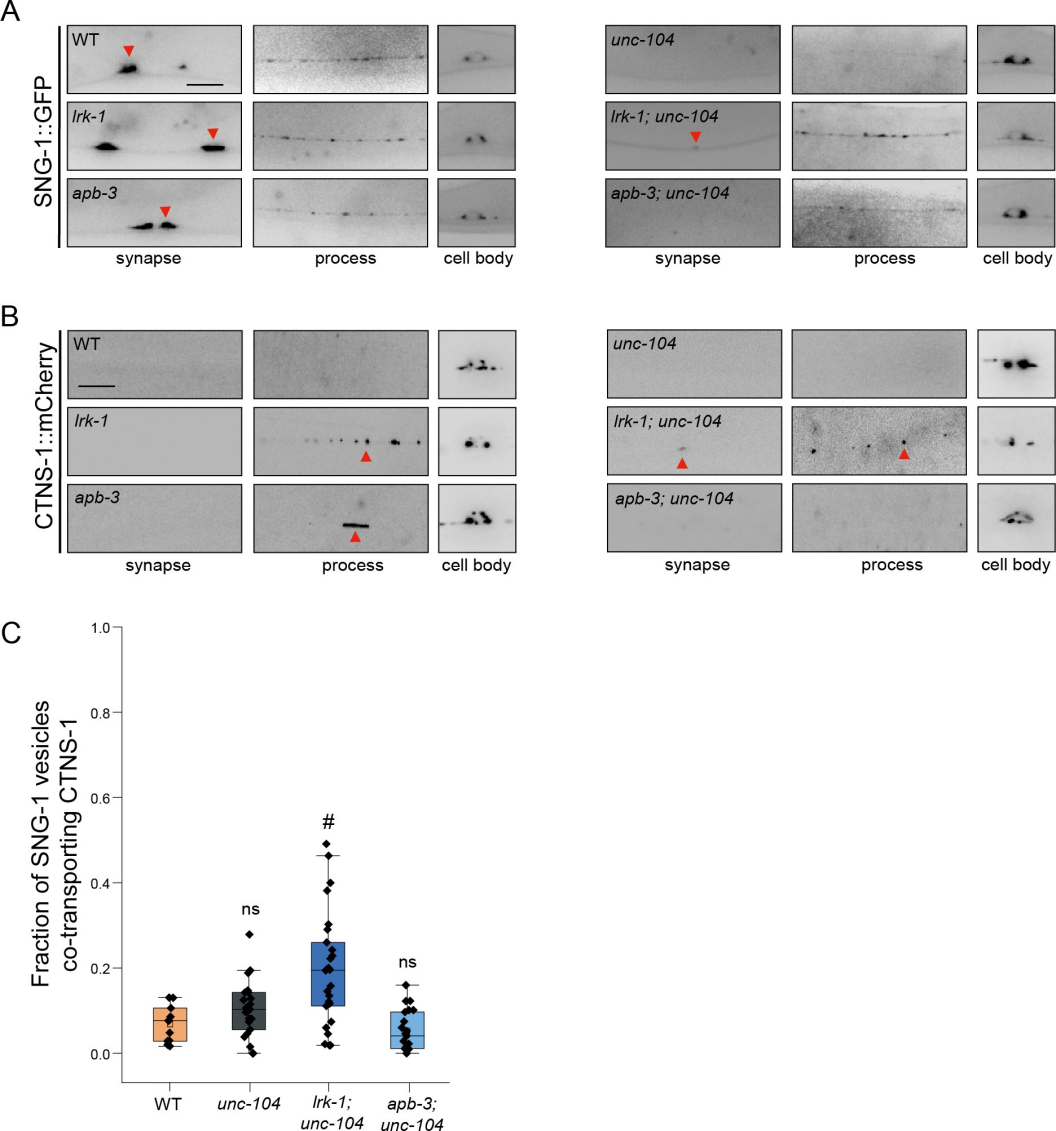
SV-lysosomes in *lrk-1* and *apb-3* mutants are dependent on UNC-104. (A) SNG-1::GFP in the cell body, process, and synapses of PLM neurons show dependence on UNC-104 in *lrk-1*(*km17*) and *apb-3*(*ok429*) mutants and their doubles with *unc-104*(*e1265tb120*). Scale bar: 10 μm. (B) CTNS-1::mCherry in the cell body, process, and synapses of PLM neurons shows dependence on UNC-104 in *lrk-1*(*km17*) and *apb-3*(*ok429*) mutants and their doubles with *unc-104*(*e1265tb120*). Red arrows highlight CTNS-1 puncta. Scale bar: 10 μm. (C) Quantitation of fraction of total moving SNG-1-carrying vesicles co-transporting CTNS-1 in *unc-104*(*e1265tb120*), *lrk-1*(*km17*)*; unc-104*, and *apb-3*(*ok429*)*; unc-104* from kymograph analysis of sequential dual color imaging done at 1.3 fps. #P-value ≤ 0.05 (Mann–Whitney Test, all comparisons to WT); ns: not significant; Number of animals (N) ≥ 18 per genotype; Number of vesicles (n) > 1200.

SNG-1 in *lrk-1* mutants is partially dependent on UNC-104, as little SNG-1 reaches the synapse in *lrk-1; unc-104* compared to that in WT and *lrk-1* mutant animals (Fig 3A). In a strong loss-of-function *unc-104* allele, RAB-3 in *lrk-1; unc-104* is shown to be partially dependent on UNC-104 [3]. However, with a weak loss-of-function *unc-104* allele, RAB-3 reaches the synapse in *lrk-1; unc-104* double mutants (S4F Fig). SNG-1- and RAB-3-carriers in *apb-3* are partially dependent on UNC-104, as both markers do not reach the synapse in *apb-3; unc-104* double mutants (Figs 3A and S4F). This suggests that SVps in *lrk-1* and *apb-3* mutants are only partially dependent on UNC-104. Additionally, RAB-3 and SNG-1 in these mutants appear to have different degree of dependence on UNC-104 (Figs 3A and S4F).

SV-lysosomes are dependent on UNC-104 for their exit from the cell body (Figs 3B, S5A and S1H, and S6 Movie). The extent of SV-lysosome localization along the neuronal process is dependent on UNC-104 in *apb-3* mutants (Figs 3B and S1H). However, similar to SVp carriers, SV-lysosomes in *lrk-1* mutants can travel in the axon independent of UNC-104 (Fig 3B and S1H). Additionally, the incidence of co-transport of CTNS-1 and SNG-1 is lowered in the *apb-3*; *unc-104* double mutant (Fig 3C), as compared to that in the *apb-3* single mutant (Fig 2B). This lowered incidence co-transport in the *apb-3*; *unc-104* double mutant likely reflects the reduction of SV-lysosomes in the axons of these mutants (Fig 3B).

SV-lysosomes in wildtype and *apb-3* mutants depend on UNC-104, suggesting that UNC-104 continues to facilitate the transport of SV-lysosomes in *apb-3* mutants. In the absence of *apb-3* more UNC-104 get recruited on to the SV-lysosomes, transporting them further into the neuronal process. SV-lysosomes in *lrk-1* mutants likely have a different balance of anterograde & retrograde motors, as well as multiple types of motors and these changes facilitate SV-lysosome anterograde transport in axons in the absence of UNC-104. As shown earlier (Figs 3A and S4F) [3], SVps in *lrk-1* and *apb-3* are only partially dependent on UNC-104 as suppression of the *unc-104* transport phenotypes by *lrk-1* mutant is not a full restoration to wildtype. This suggests that, unlike in wildtype, multiple anterograde motors transport SVps in *lrk-1* and *apb-3* mutants.

### SV-lysosomes in *lrk-1* and *apb-3* mutants are differentially dependent on SYD-2

The active zone protein SYD-2 has been shown to regulate lysosomal protein distribution in *unc-16* mutants in *C*. *elegans* neurons [45]. SYD-2 is also a known genetic enhancer of UNC-104 and is known to directly bind this motor (27–30). Further, SYD-2 is thought to be critical for UNC-104 motor clustering [46–48]. Since SV-lysosome transport depends on UNC-104, we used a null allele *syd-2*(*ok217)*, to test whether the altered trafficking of the SV-lysosomal compartments in *lrk-1* and *apb-3* also depends on SYD-2 [29].

**Fig 4:**
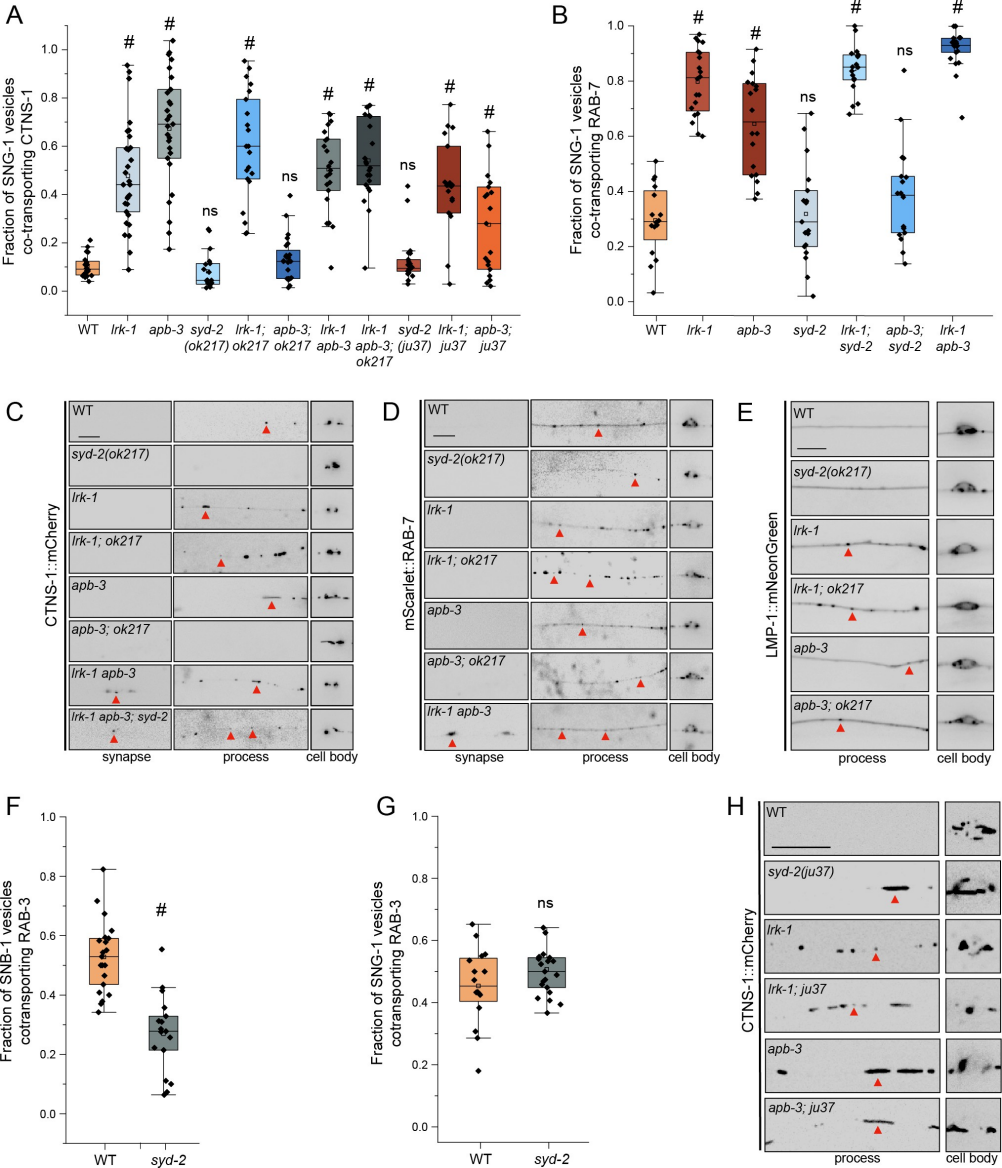
Distribution of SV-lysosomal compartments depends on UNC-104. (A) Quantitation of fraction of total moving SNG-1-carrying vesicles co-transporting CTNS-1 in *syd-2* mutants and their doubles with *lrk-1*(*km17*) and *apb-3*(*ok429*), from kymograph analysis of dual color imaging. *ok217* refers to the null allele of *syd-2*, *syd-2*(*ok217*), while *ju37* refers to the *syd-2*(*ju37*) allele. #P-value ≤ 0.05 (Mann–Whitney Test, all comparisons to WT); ns: not significant; Number of animals (N) ≥ 18 per genotype; Number of vesicles (n) > 750. Values for *lrk-1* and *apb-3* single mutants are the same as those in Fig 2B. (B) Quantitation of fraction of total moving SNG-1-carrying vesicles co-transporting RAB-7 in *syd-2*(*ok217*) and its doubles with *lrk-1*(*km17*) and *apb-3*(*ok429*), from kymograph analysis of dual color sequential imaging at 1.3 fps. P-value > 0.05 (One-Way ANOVA with Tukey’s post-hoc test); ns: not significant; N ≥ 19 per genotype; n > 700. Values for *lrk-1* and *apb-3* single mutants are the same as those in Fig 2C. (C) CTNS-1::mCherry in the cell body, process, and synapses of PLM neurons of *syd-2*(*ok217*) mutant and its doubles with *lrk-1*(*km17*) and *apb-3*(*ok429*). Red arrows highlight some CTNS-1-carrying compartments, some fainter. Scale bar: 10 μm. (D) mScarlet::RAB-7 in the cell body, process, and synapses of PLM neurons of *syd-2*(*ok217*) mutant and its doubles with *lrk-1*(*km17*) and *apb-3*(*ok429*). Red arrows highlight some RAB-7-carrying compartments, some fainter. Scale bar: 10 μm. (E) LMP-1::mNeonGreen in the cell body, process, and synapse of PLM neurons of *syd-2*(*ok217*) mutant and its doubles with *lrk-1*(*km17*) and *apb-3*(*ok429*). Red arrows indicate LMP-1-carrying compartments. Scale bar: 10 μm. (F) Quantitation of fraction of total moving SNB-1-carrying vesicles co-transporting RAB-3 in WT and *syd-2*(*ok217*), from simultaneous dual color imaging at 3 frames per second (fps). # P-value ≤ 0.05 (2 tailed Student’s t test); N > 20. (G) Quantitation of fraction of total moving SNG-1-carrying vesicles co-transporting RAB-3 in WT and *syd-2*(*ok217*) from sequential dual color imaging at 1.3 fps. P-value > 0.05 (One-Way ANOVA with Tukey’s post-hoc test); ns: not significant; N > 15. (H) CTNS-1::mCherry in the cell body, process, and synapses of PLM neurons of *syd-2*(*ju37*) mutant and its doubles with *lrk-1*(*km17*) and *apb-3*(*ok429*). Red arrows highlight some CTNS-1-carrying compartments, some fainter. Scale bar: 10 μm. Imaged at 100×.

Trafficking of SV-lysosomes, measured by the co-transport of SVps such as SNG-1, SNB-1 and RAB-3, with CTNS-1 and RAB-7, is not affected in *syd-2(ok217)* mutants (Figs 4A, 4B and S5A–S5C). The extent of localization of the SV-lysosomes in *syd-2* mutant neuronal processes is somewhat reduced as compared to WT animals (Figs 4C and 4D, S1H, S1I and S1K and S7 Movie). Similarly to what we observed with *unc-104* mutants, trafficking of SV-lysosomes, measured by both the co-transport of SNG-1 and CTNS-1 and the localization of CTNS-1 and LMP-1 in the PLM neuronal process, in *lrk-1* mutants is largely independent of SYD-2 (Figs 4A–4E and S1H–S1J). However, the SV-lysosomal trafficking in *apb-3* mutants is dependent on SYD-2 (Figs 4A–4D and S1H–S1I). Thus, SYD-2 and UNC-104 have similar effects on the localization of SV-lysosomes. As the *apb-3* phenotypes appear to be dependent on UNC-104 (Fig 3B) and SYD-2, it is likely that *syd-2* acts downstream of *apb-3* to facilitate UNC-104-dependent transport of SV-lysosomes. SYD-2 likely functions to cluster UNC-104 on SV-lysosomes in *apb-3* mutants. Further, SV-lysosome trafficking phenotypes in triple mutants of *lrk-1 apb-3*; *syd-2* are similar to those in the *lrk-1 apb-3* double mutants (Figs 4A, 4C and S1H), suggesting a hierarchical pathway wherein LRK-1 acts upstream of the AP-3 complex, and SYD-2 acts downstream of AP-3 to likely facilitate UNC-104-dependent transport.

In order to examine whether SYD-2 affects trafficking of SV proteins like LRK-1 and AP-3 [3], we examined the co-transport of SNB-1 and SNG-1 with RAB-3. The incidence of cotransport of SNB-1 with RAB-3 is significantly lowered in *syd-2*(*ok217*) mutants (~15%) than in wildtype (~35%) (Fig 4F), similar to that reported in *lrk-1* and *apb-3* single mutants [3]. Furthermore, *syd-2(ok217)* mutants, like *lrk-1* and *apb-3* mutants, do not affect the co-transport of SNG-1 with RAB-3 (Fig 4G). These observations suggest that SYD-2 only affects the trafficking of a subset of SVps and is consistent with its role being downstream of AP-3.

We next examined APB-3::GFP localization in *syd-2(ok217)* mutants and observed many more and brighter APB-3::GFP puncta, suggesting that AP-3 may show persistent association with membranes (Figs 2G–2J and S4E). We think that SYD-2 may not influence the ability of the AP-3 complex to associate with membrane surfaces, but rather facilitates the transport of compartments formed after AP-3 has assembled on them.

### N-terminus is sufficient for the presence of SV-lysosomes along the neuronal process in *apb-3* mutants

The SYD-2/ Liprin-α N- and C-terminal regions both bind to KIF1A/UNC-104 [28–30], and have intermolecular interactions with one another important for SYD-2’s role in synapse formation [49,50]. The loss-of-function allele *syd-2*(*ju37*) encodes a part of the N-terminal region of SYD-2 that is known to physically associate with UNC-104 [28–30]. We therefore used this allele to examine if the SYD-2 N terminal region is sufficient for altering the SV-lysosomal phenotypes observed in *lrk-1* and *apb-3* mutants.

The extent of co-transport of SNG-1 and CTNS-1 in *syd-2*(*ju37*) is similar to that in WT and *syd-2*(*ok217*) (Fig 4A). However, SV-lysosomes appear to transport farther into the neuronal processes of *syd-2*(*ju37*) mutants (Figs 4H and S1H). Further, *syd-2*(*ju37*), like *syd-2*(*ok217*) does not alter trafficking of SV-lysosomes in *lrk-1* mutants. However, unlike in *apb-3*; *syd-2*(*ok217*) double mutants, SV-lysosomes in *apb-3*; *syd-2*(*ju317*) double mutants are transported out of the cell body (Figs 4A, 4H and S1H). These data suggest that the SYD-2 N terminal region is sufficient to enable transport of SV-lysosomes in WT and *apb-3* mutants.

Since multiple domains of SYD-2 physically associate with UNC-104, we assessed the roles of these domains in the distribution of SV-lysosomes in *apb-3* mutant animals. To this end, we expressed a series of SYD-2 constructs, each lacking a specific domain, in *apb-3* mutants (S5D and S5E Fig). Transgenic expression of full length (FL) SYD-2 appears to drive SV-lysosomes farther into the neuronal processes of WT animals, suggesting that SV-lysosomal transport is likely sensitive to levels of intracellular SYD-2. Both the construct expressing the N-terminal 719 aa of SYD-2 and the construct lacking SAM domains (SYD-2 Δ SAM) do not suppress the *apb-3* SV-lysosome phenotype as the CTNS-1 compartments continue to come out along the neuronal process (S5F and S5G Fig). Both these regions have also been reported to physically interact with UNC-104 [28,30,51]. Therefore, these data are consistent with our earlier data suggesting that the SYD-2 N terminal region is sufficient to enable transport of SV-lysosomes (Figs 4A, 4E and S1H). However, complex intra-molecular interactions are likely involved in SYD-2 function, similar to that reported for the synaptic development roles of SYD-2 [49,52].

Thus, the N-terminus of SYD-2 appears to be sufficient to facilitate SV-lysosome transport in *apb-3* mutants. Since the N-terminus of SYD-2 is important for its role at the active zone as well as binding to the KIF1A motor, it is difficult to assess if the *syd-2*(*ju37*) phenotypes are solely dependent on its ability to partially retain binding to UNC-104.

### SYD-2 facilitates UNC-104-depenedent transport of SVp carriers

The known physical and genetic interactions between SYD-2 and UNC-104 suggest that SYD-2 may facilitate cargo transport through UNC-104 [27,29]. Therefore, we examined the potential role of the UNC-104–SYD-2 complex in the localization and transport of SVp carriers and SV-lysosomes. We used two cargo-binding defective alleles of *unc-104*, the stronger *unc-104(e1265)* and the weaker *unc-104(e1265tb120)* [44].

**Fig 5:**
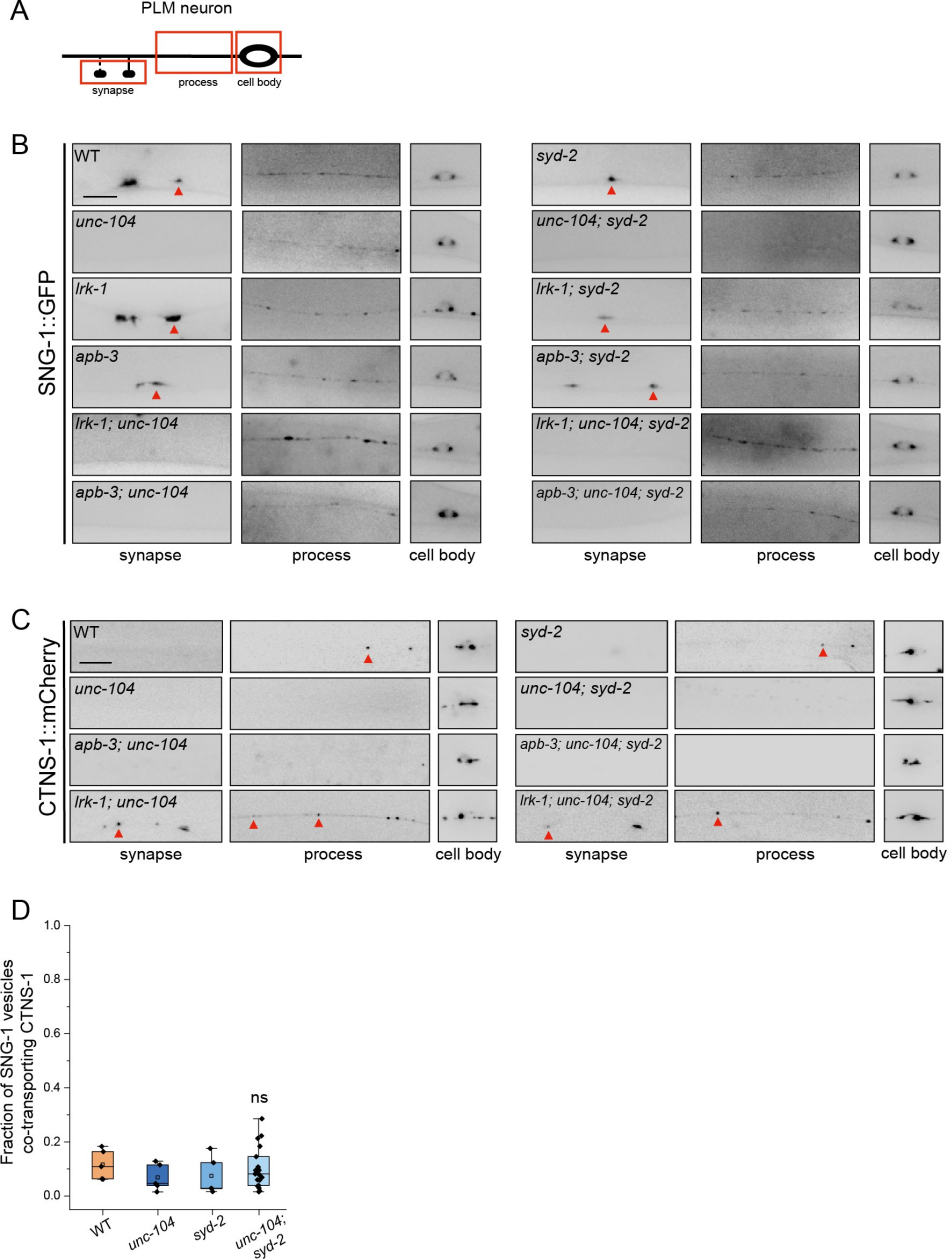
SYD-2 is required for UNC-104 dependent of SVp carriers. (A) Schematic of the PLM neuron. The red box highlights the region of imaging. (B) SNG-1::GFP in the cell body, process and synapses of PLM neurons of *syd-2*(*ok217*) and *unc-104*(*e1265tb120*), and their doubles with *lrk-1*(*km17*) and *apb-3*(*ok429*). Scale bar: 10 μm. (C) CTNS-1::mCherry in the cell body, process and synapses of PLM neurons of *syd-2*(*ok217*) and *unc-104*(*e1265tb120*), and their doubles with *apb-3*(*ok429*). Scale bar: 10 μm. (D) Quantitation of fraction of total moving SNG-1-carrying vesicles co-transporting CTNS-1 in WT, *unc-104*(*e1265tb120*), *syd-2*(*ok217*), and *unc-104*; *syd-2* from kymograph analysis of sequential dual color imaging at 1.3 fps. P-value > 0.05 (Mann–Whitney Test, all comparisons to WT); ns: not significant; Number of animals (N) > 20 for *unc-104; syd-2*; Number of vesicles (n) >1000.

In wildtype animals, the trafficking to the synapse of the transmembrane SVps SNG-1, SNB-1, and the peripheral membrane protein RAB-3 is dependent on UNC-104 but not on SYD-2 (Figs 5B and S6A–S6D). However, SVps travel less distance in *unc-104; syd-2* than in *unc-104* single mutants, demonstrating that SYD-2 facilitates UNC-104-dependent SVp transport (Figs 5B and S6A–S6D) [27]. SNG-1-carrying vesicles are transported to similar extents in *lrk-1; unc-104* double and *lrk-1; unc-104; syd-2* triple but the transport of RAB-3-carrying vesicles appears to be partially dependent on SYD-2 (Figs 5B, S4F, S6B and S6D). Using the stronger loss of function allele of *unc-104*, *unc-104*(*e1265*), we see that both SNG-1- and SNB-1-carrying vesicles in *lrk-1; unc-104(e1265)* double mutants are partially dependent on UNC-104 and SYD-2 (S6B and S6D Fig). Likewise, *apb-3; unc-104* and *apb-3; unc-104; syd-2* show comparable localization of both SNG-1 and RAB-3 along the neuronal process (Figs 5B, S4F, S6B and S6D). Using *unc-104(e1265)*, we see that both SNG-1- and SNB-1-carrying vesicles in *apb-3; unc-104(e1265)* double mutants are partially dependent on UNC-104 and independent of SYD-2 (S6B and S6D Fig). Together, these data indicate that SYD-2 facilitates UNC-104-dependent SVp transport in *lrk-1* but not in *apb-3* mutants.

SV-lysosomes, marked by CTNS-1, are dependent on UNC-104 and largely independent of SYD-2 in wildtype (S5A Fig), as demonstrated by the localization of CTNS-1 as well as the extent of co-transport of SNG-1 and CTNS-1, which are both similar to WT in *unc-104*, *syd-2* single and *unc-104; syd-2* double mutant animals (Fig 5D). *lrk-1; unc-104* double and *lrk-1; unc-104; syd-2* triple mutants show comparable localization of CTNS-1 along the neuronal process (Figs 5C and S1H), suggesting that SYD-2 does not facilitate UNC-104-dependent transport of SV-lysosome in *lrk-1* mutants. The SV-lysosomes in *apb-3* mutants are dependent on both UNC-104 and SYD-2 (Figs 3B, 5C and S1H), suggesting in *apb-3* mutants, the UNC-104-SYD-2 complex no longer preferentially transports SVp carriers and instead transports SV-lysosomes.

Since these data suggest that SYD-2 functions to facilitate UNC-104-dependent transport, we examined whether SYD-2 affects the recruitment of UNC-104 onto SVp carriers. To this end, we measured the intensity and flux of moving UNC-104::GFP in the region of *Ce* neurons proximal to the cell body (S6E and S6F Fig) [53]. Both the intensity and flux of UNC-104::GFP were comparable in WT and *syd-2*(*ok217*) mutants (S6G and S6H Fig). However, run lengths of SNG-1-carrying SVp carriers are significantly lowered in *syd-2* mutants when compared to WT (S5I and S5J Fig). Together, these data suggest that SYD-2 likely does not affect the recruitment of UNC-104 onto the cargo surface, but functions to cluster/activate UNC-104 to influence cargo transport.

### SYD-2 and the AP-1 complex together regulate the polarized distribution of SVps

**Fig 6:**
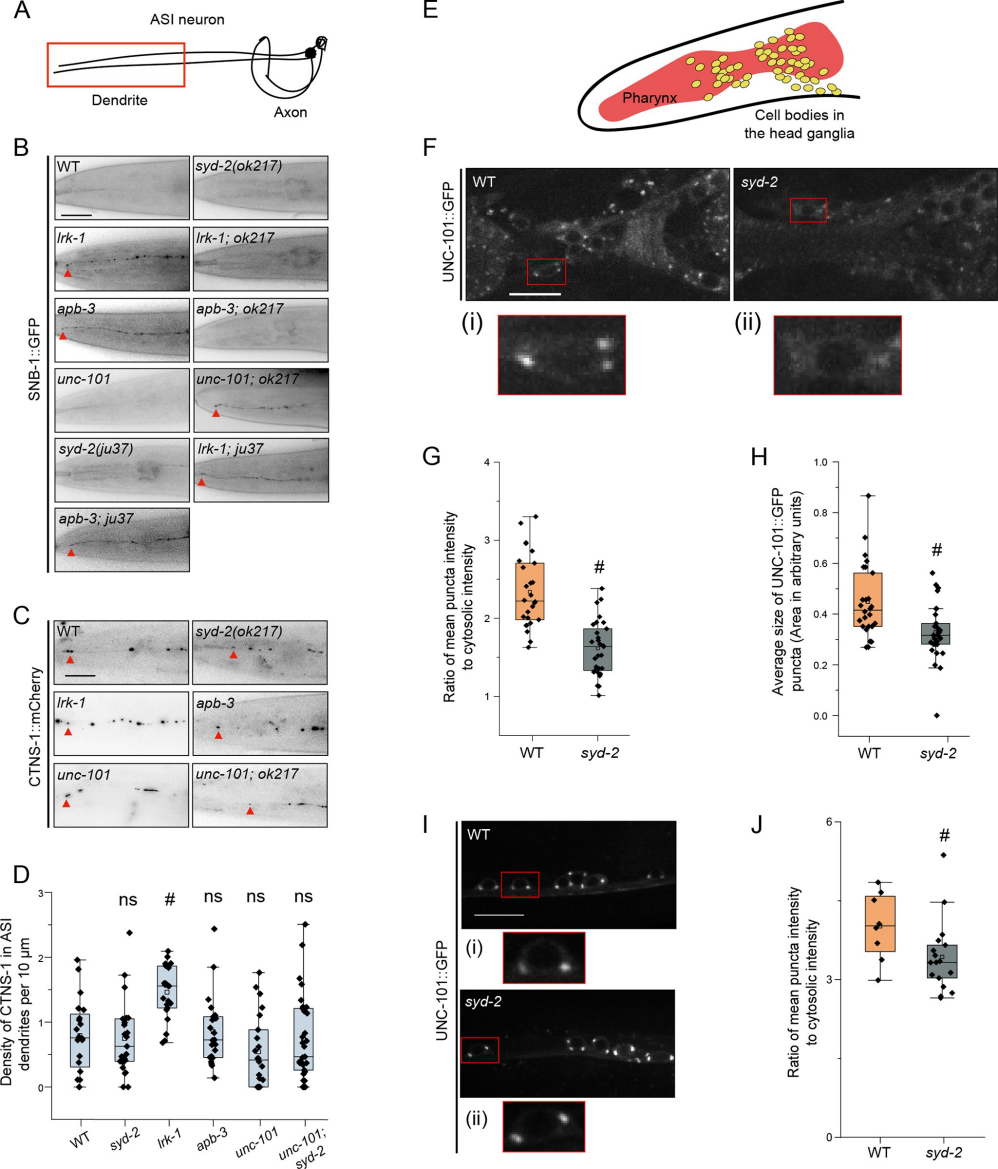
SYD-2 and the AP-1 complex together regulate the polarized distribution of SVps to axons. (A) Schematic of the ASI chemosensory neuron. Red box highlights the region of imaging. (B) SNB-1::GFP in the dendrite of the ASI neuron of WT and two alleles of *syd-2* and their doubles with *lrk-1*(*km17*) and *apb-3*(*ok429*). *ok217* represents *syd-2*(*ok217*) allele. *ju37* represents *syd-2*(*ju37*) allele. *unc-101*(*m1*) is a substitution mutation in the μ chain of the AP-1 complex causing a premature stop. Red arrows point to the SNB-1::GFP signal at the dendrite tip. Scale bar: 20 μm. Number of animals (N) > 6 per all single mutant genotypes; N > 20 for all double mutant genotypes. (C) CTNS-1::mCherry in the dendrite of the ASI neurons of WT, *syd-2*(*ok217*), *lrk-1*(*km17*), *apb-3*(*ok429*), *unc-101*(*m1*), and *unc-101*(*m1*)*; syd-2*(*ok217*). Red arrows point to CTNS-1 compartments in the dendrite. Scale bar: 20 μm. N > 20 per genotype. (D) Density (number of CTNS-1 puncta per 10 μm in the ASI dendrite) of CTNS-1 in the ASI dendrite. # P-values ≤ 0.05 (Mann–Whitney Test, black comparisons against WT and blue comparisons against *lrk-1*); N > 20 for each genotype. (E) Schematic of *C*. *elegans* head shows the pharynx (red) and the head ganglion cell bodies (yellow). (F) Images show UNC-101::GFP puncta in the head ganglion cell bodies of WT and *syd-2*(*ok217*). Scale bar: 10 μm. The red boxes highlight the regions of insets with cell bodies from images show UNC-101::GFP in (i) WT and (ii) *syd-2*. (G) Quantitation of intensity of UNC-101::GFP puncta in the head ganglion cell bodies in WT and *syd-2*(*ok217*). The ratio of the intensity of UNC-101::GFP puncta to cytosolic intensity in the cell body is plotted. # P-value ≤ 0.05 (One-Way ANOVA with Tukey’s post-hoc test); N > 5 animals; n > 25 cell bodies. (H) Quantitation of average size of UNC-101::GFP puncta per cell body in WT and *syd-2*(*ok217*). # P-value ≤ 0.05 (Mann–Whitney Test); N > 5 animals; n > 25 cell bodies. (I) Images show UNC-101::GFP puncta in the cell bodies of the ventral nerve cord neurons in WT and *syd-2*(*ok217*). Scale bar: 10 μm. The red boxes highlight the regions of insets with cell bodies from images show UNC-101::GFP in (i) WT and (ii) *syd-2*. (J) Quantitation of intensity of UNC-101::GFP puncta in the cell bodies of the ventral nerve cord in WT and *syd-2*(*ok217*). The ratio of the intensity of UNC-101::GFP puncta to cytosolic intensity in the cell body is plotted. # P-value ≤ 0.05 (Mann–Whitney test); N > 5 animals; n > 10 cell bodies.

SNB-1-labeled SVp carriers in *lrk-1* and *apb-3* mutants mislocalize to dendrites [3,41]. Since *syd-2* mutants phenocopy *lrk-1* and *apb-3* mutants in affecting the co-transport of SNB-1 and RAB-3 (Fig 4F) [3], we examined whether SNB-1 mislocalizes to dendrites in *syd-2* mutants. Similar to wildtype, SNB-1 was found to be excluded from the dendrites of the ASI neuron in *syd-2*(*ok217*) mutants (Fig 6B and Table 1), which show a similar orientation of axonal and dendritic microtubules as wildtype (S7A Fig). Thus, SYD-2 by itself does not appear to play a key role in regulating polarized trafficking of SVps. However, SNB-1-labeled SVp carriers in *lrk-1* and *apb-3* mutants are dependent on SYD-2 for their dendritic mislocalization (Fig 6B).

**Table 1:** Extent of SNB-1 presence in the dendrite of ASI neuron.

Genotype	Average % length of dendrite showing SNB-1 signal	Standard deviation
WT	39%	27
*lrk-1*(*km17*)	92%	2
*apb-3*(*ok429*)	69%	28
*syd-2*(*ok217*)	25%	25
*lrk-1*(*km17*)*; syd-2*(*ok217*)	35%	23
*apb-3*(*ok429*)*; syd-2*(*ok217*)	45%	28

Previous studies have shown that the dendritic mislocalization of SNB-1 in *lrk-1* depends on the UNC-101/μ subunit of the AP-1 complex [3,41]. Therefore, we examined whether *syd-2* genetically interacts with *unc-101* to regulate the polarized distribution of SVps. *unc-101; syd-2*(*ok217*) double mutants fail to exclude SNB-1 from the ASI dendrite (Fig 6B and Table 1), suggesting that SYD-2 acts similarly to the AP-1 complex to facilitate SVp entry into dendrites in *lrk-1* and *apb-3* mutants. However, unlike the null allele *syd-2*(*ok217*), the loss-of-function allele *syd-2*(*ju37*) does not suppress the dendritic mislocalization of SNB-1 to the ASI dendrite in *lrk-1* and *apb-3* mutants (Fig 6B).

We next assessed CTNS-1 localization in dendrites and found that only *lrk-1* shows a significant increase in the number of dendritic CTNS-1 puncta, while *apb-3*, *syd-2*(*ok217*), *unc-101*, and *unc-101; syd-2* are all similar to wildtype (Fig 6C and 6D). These data suggest that SYD-2 specifically regulates the polarized distribution of SVps.

To assess whether SYD-2 interacts with UNC-101 to regulate other UNC-101 functions, we first assessed whether localization of the dendritic receptor ODR-10, which is known to depend on UNC-101 [54]. *unc-101* mutants show mislocalization of the ODR-10::GFP receptor to the AWC axon, while *syd-2* mutants do not (S7B Fig). We have previously shown that UNC-101 regulates the length of the SVp carriers that exit the neuronal cell bodies [3]. *unc-101* mutants form longer SVp carriers than that seen in wildtype; however, *syd-2* does not alter the longer SVp carrier length seen in *unc-101* mutants (S7C Fig).

These data suggest that SYD-2 genetically interacts with the AP-1 complex only to prevent SVp entry into dendrites and thus is not a general interactor of AP-1. Additionally, the SYD-2 N-terminus is likely sufficient to enable dendritic entry of atypical SVp carriers formed in *lrk-1* and *apb-3* mutants. Furthermore, *lrk-1* seems to have wider dendritic trafficking defects than those seen in *apb-3*.

### SYD-2 alters the localization of UNC-101

Since *syd-2* acts like *unc-101* in preventing dendritic localization of SNB-1 in *lrk-1* and *apb-3* mutants, we examined if *syd-2* alters the localization of UNC-101::GFP in the cell bodies of neurons in the head (Fig 6E). The UNC-101::GFP puncta are fainter and smaller in *syd-2* mutants (Fig 6F–6H), and more cell bodies have no or fewer UNC-101::GFP puncta in the head neurons of *syd-2* mutants compared to that in wildtype (S7D Fig). In ventral cord neurons too *syd-2* affects the intensity of UNC-101::GFP puncta (Fig 6I and 6J). Further, the loss-of-function allele of *syd-2*, *syd-2*(*ju37*), did not alter the intensity or size of UNC-101::GFP puncta (S7E–S7G Fig). This suggests that the SYD-2 N-terminus is sufficient for AP-1 localization.

Thus, SYD-2 regulates the localization of UNC-101 in *C*. *elegans* neurons, which might account for its role in suppressing the dendritic mistrafficking of SVps in head neurons.

## Discussion

**Fig 7:**
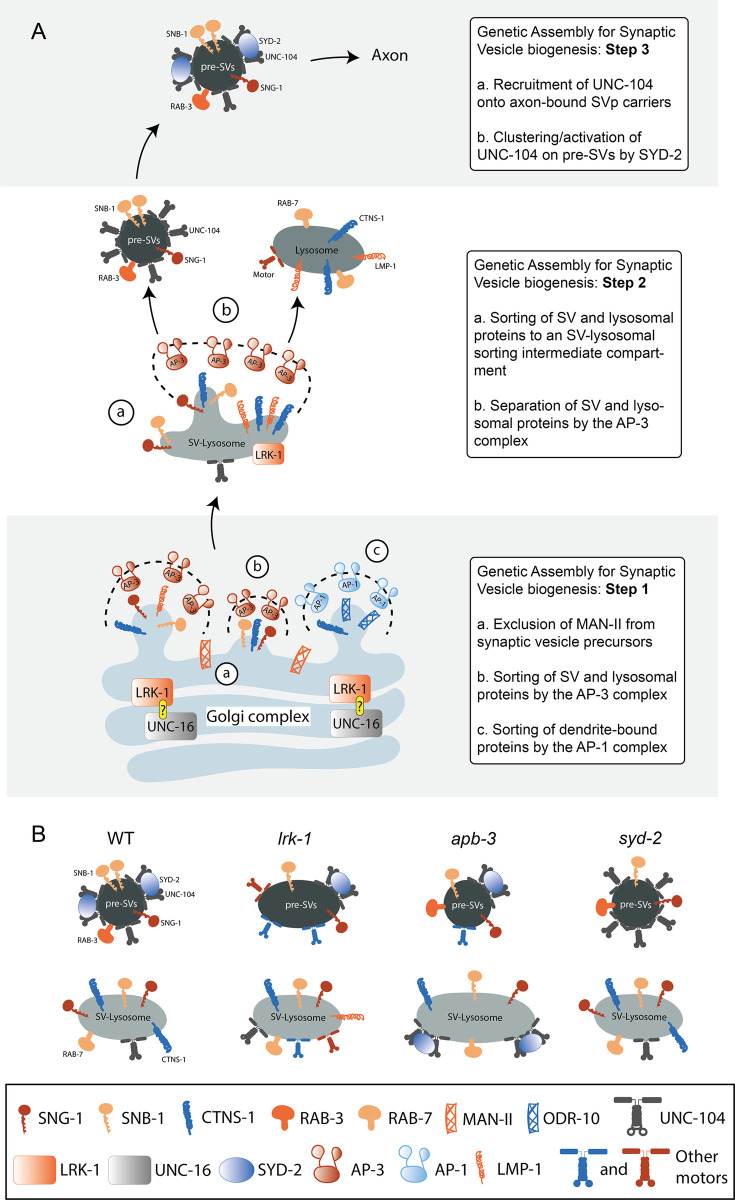
SYD-2 facilitates UNC-104-dependent transport of SVp carriers formed by LRK-1 and the AP-3 complex. (A) Three hierarchical trafficking steps for formation of SVp carriers and lysosomes. LRK-1 acts with UNC-16 at the Golgi to regulate trafficking of SVp and lysosomal proteins through regulation of the localization of both AP-1 and AP-3 complexes. The question mark LRK-1 and UNC-16 represents a physical interaction between LRK-1 and UNC-16. LRK-1 acts via the AP-3 complex to facilitate formation of the post-Golgi intermediate compartments, SV-lysosomes, from which SVps and lysosomal proteins likely separate into SVp carriers and lysosomes. SYD-2 facilitates UNC-104 dependent transport of SVp carriers into the axon. (B) Schematic representation of SVp carriers and SV-lysosomes formed in different genotypes.

Our study, like others, shows that SVps are trafficked in many heterogenous carriers and sometimes with lysosomal proteins, suggesting that SVps and lysosomal proteins share trafficking routes (Figs 1A–1C and 2A–2C) [10,14,15]. The small fraction of vesicles co-transporting SV and lysosomal proteins, the SV-lysosomes, are a trafficking intermediate in the formation of SV precursors and lysosomes (Fig 7A). The transport of SV-lysosomes, like SVp carriers, also depends on UNC-104 (Figs 3B, S1H and S5A). Although our work has the limitation of using primarily overexpression-based transgenic lines to assess the composition of transport carriers, some of our observations in wild type neurons are corroborated by recent studies examining the trafficking of SVps in mammalian systems using CRISPR knock-in reporter lines [55].

LRK-1 and the AP-3 complex, help in sorting SVps and lysosomal proteins (Fig 2B and 2C) [14]. In addition, *lrk-1* mutant animals appear to have more widespread trafficking defects of lysosomal proteins in comparison to *apb-3* mutants (Fig 2F). UNC-104 requires SYD-2 to facilitate the transport of SVp carriers that lack lysosomal proteins in wildtype (Figs 5B, S4F, and S6A–S6D) [27]. SV-lysosomes are dependent on UNC-104, but are not as strongly dependent on SYD-2 (Figs S1H, S1K and S5A). However, in the absence of the AP-3 complex, the preference is switched such that the SV-lysosomes depend on both UNC-104 and SYD-2, but the SVs are partially dependent only on UNC-104 and independent of SYD-2 (Figs 3A, 3B, 5B, 5C and S4F). This might reflect the increased number of UNC-104 on SV-lysosomes in *apb-3* mutants and fewer UNC-104 motors on the SVp carriers lacking lysosomal proteins. Some effects on SYD-2 are also likely to be mediated via AP-3 localization to membrane surfaces, perhaps working in concert with UNC-104 to regulate the kinetics of AP-3 membrane cycling. Finally, SYD-2 appears to have roles with another clathrin adaptor complex, AP-1. The polarized trafficking of SVps appears to require either SYD-2 or UNC-101, which act redundantly with each other likely due to the role of SYD-2 in enabling localization of the AP-1 complex to the Golgi (Fig 6F).

LRRK2 is known to affect the trafficking of lysosomal proteins [42,56,57], SVps [3,41,58], retromer and ER-Golgi proteins [59–61], dense core vesicle proteins [57], RAB GTPases [62–64], neurotransmitter transporters [65], autophagy-related proteins LC3 and LAMP-1 [66], and mitochondria [67]. The trafficking and localization of lysosomal proteins via LRRK2 seem to depend on RAB-7 and the retromer complex [68–70]. AP-3 is also known to play a key role in sorting lysosomal proteins in a variety of cells and separating SVps from lysosomal proteins at a common trafficking compartment [2,14,56]. The *C*. *elegans* AP-3 complex is shown to be a downstream effector of LRK-1/LRRK2 in axon outgrowth and the co-transport of SNB-1 and RAB-3 along the neuronal process [3,56].

*lrk-1* mutants affect the trafficking of more widespread trafficking defects than *apb-3* mutants, such as the presence of LMP-1 along the neuronal process and the presence of CTNS-1 in the dendrite (Figs 2F and 6C), while *lrk-1* and *apb-3* mutants appear to share all other remaining phenotypes [56,71,72], notably that many more SNG-1-transport carriers contain CTNS-1 (S3B Fig). The AP-2 complex is reported to regulate the trafficking of LAMP-1 and LAMP-2 to the lysosomes via the plasma membrane, while the AP-3 complex has little effect on their trafficking [72]. This supports our data that LMP-1 trafficking is likely mediated by LRRK2 independently of the AP-3 complex. Thus, LRK-1 may act upstream of AP-3; however, our data do not fully exclude the possibility that LRK-1 and AP-3 act additively to regulate the localization of a subset of lysosomal markers (S1H–S1J Fig).

The AP-3 complex can physically bind to LRRK2 [56,71]. Therefore, some of the trafficking defects seen in *lrk-1* may occur through its ability to affect the efficient recruitment of the AP-3 complex to membrane surfaces (Fig 2G–2J), as has already been seen for AP-1 [3]. Phosphorylation of the AP-3 complex has been shown to be necessary to recruit on SVs and to play a role in endosomal SV biogenesis [73]. Further, LRRK2 has been shown to physically interact with the AP-2 complex via its ROC domain [71]. The LRRK2 ROC domain regulates the LRRK2 kinase activity [74]. Therefore, LRRK2, via its kinase activity [71], could regulate the localization or activity of the AP-3 complex. Alternatively, LRK-1 could alter the composition of membrane compartments [42] and indirectly affect the recruitment and function of the AP-3 complex.

We believe that SV and lysosomal proteins share early trafficking routes and may emerge together from the Golgi complex. The SV-lysosomes could be (a) post-Golgi intermediate compartments from which SV and lysosomal proteins are sorted into precursors of synaptic vesicles (pre-SVs) and lysosomes by LRK-1 and AP-3 (Fig 7A) [75]. In *lrk-1* and *apb-3* mutants, the population of SVp carriers that transport both SVps and lysosomal proteins increases as a consequence of defective early sorting of SV and lysosomal proteins. Alternatively, missorting in *lrk-1* and *apb-3* mutants simply leads to these markers getting sorted onto vesicles that use other motors and thereby are able to get out.

UNC-104/KIF1A is a critical motor for transporting SVps [11,18,20]. The SV-lysosomes in wildtype, although dependent on UNC-104, do not extend very far into the axon (Figs 3C and S1H), perhaps because they have fewer numbers of UNC-104 motors on their surface compared to SVp carriers lacking lysosomal proteins (Fig 7B and Table T in S1 Text). In *lrk-1* and *apb-3* mutants, SVs partially depend on UNC-104 (Figs 3A and S6A–S6D) [3]. In *lrk-1* mutants, both SVs and SV-lysosomes likely depend on multiple motors for their axonal transport, much like that seen in *unc-16* mutants, where UNC-16 acts upstream of LRK-1 [3,76,77] (Fig 7A and 7B and Table T in S1 Text). LRK-1 appears important to ensure that both SVs and SV-lysosomes are dependent on UNC-104. Thus, appropriate sorting by LRK-1 may ensure that sufficient numbers of UNC-104 motors are present on trafficking precursors containing SVps. The action of AP-3 seems critical to ensure that the UNC-104-SYD-2 complex acts predominantly on SVp carriers lacking lysosomal proteins. The transport of SVps in *lrk-1* and *apb-3* likely depend on other motors that are recruited owing to the altered compositions arising from sorting defects of SVps and lysosomal proteins. However, *syd-2* mutants, despite sharing some SVp trafficking defects with *lrk-1* and *apb-3* mutants (Fig 4F and 4G), retain UNC-104 dependence for both SV and SV-lysosome transport, suggesting that SYD-2 is unlikely to aid in recruiting UNC-104 on the cargo surface (Figs 5B, 5C and S4F). We believe that the roles for SYD-2 are through UNC-104 as previously suggested [27,51] as SVps localize to the PLM synapses in *syd-2* mutants but show altered motion properties (Figs 5B, S6I and S6J), suggesting that SYD-2 likely clusters/activates UNC-104 on the cargo surface.

Active zone proteins like Piccolo and Bassoon have been thought to cluster vesicles, and some studies suggest that such active zone proteins can be transported in carriers along with SVps [78–80]. SYD-2 is both an UNC-104 interactor and an active zone protein [27,81]. SYD-2 is known to physically associate with the motor, cluster UNC-104, and regulate motor processivity [27–30]. Additionally, several proteins are known to modulate SYD-2-dependent regulation of the UNC-104 motor [46–48]. The SYD-2-mediated clustering of UNC-104 and increase in processivity might account for UNC-104 SVp transport dependence on SYD-2. The effect of SYD-2 on UNC-104-dependent transport may rely on the pre-existing numbers of UNC-104 recruited on the cargo surface. A larger number of motors on the cargo surface may be more sensitive to the UNC-104-clustering activity of SYD-2. *syd-2* mutants continue to transport SVps to synapses. The mutants show a small reduction in the extent of localization of lysosomal proteins along the neuronal process but not their degree of co-transport with SVps (Figs 4A–4G, 5B, S4A–S4C and S4F). Additionally, SYD-2 does not affect the recruitment of UNC-104 onto cargo surface or UNC-104 motion properties (S6G and S6H Fig), but SYD-2 does affect the ability of SVp cargo to move processively as evidenced by the reduced run lengths of SNG-1-positive vesicles in *syd-2* mutants (S6I and S6J Fig). This suggests that SYD-2, despite interacting with UNC-104, does not have major roles in the transport or localization of UNC-104 by itself, but affects the transport of UNC-104-dependent cargo. The reduction in the transport of SV-lysosomes in *apb-3* depends on the presence of an UNC-104-interacting domain of SYD-2 (Fig 4A; note *apb-3; syd-2*(*ju37*), S5F and S5G Fig). Several regions of SYD-2 can bind to UNC-104, but it appears that the N-terminal region of SYD-2 can seems necessary to regulate transport of SV-lysosomes over the C-terminal region (S5F and S5G Fig). However, given that the two N-terminal constructs N719 and N517 seem different in their requirement for transport of SV-lysosomes, it is likely that a part of the IDR domain is likely also necessary for SV-lysosomal transport. However, these interactions are not very clear and it is possible that multiple domains of SYD-2 and possibly complex intramolecular interactions are needed for SV-lysosomal transport in *apb-3* mutants (S5F and S5G Fig). In the absence of SYD-2’s UNC-104-interacting domains, UNC-104 may not effectively cluster on the surface of SV-lysosomes and therefore transport of these compartments is reduced. Thus, our data can be explained by SYD-2’s action with UNC-104 rather than a role in clustering multiple vesicles. A role of SYD-2 via regulating the balance/activity of microtubule-dependent motors has also been proposed in lysosome localization in motor neurons of *C*. *elegans* [32]. Some of these phenotypes may also arise from changes in levels of expression of SV proteins in *syd-2* mutants [81].

Localization of the AP complexes is altered in *syd-2* mutants (Figs 2G–2J, 6F–6H and S4E, S7D). There are more and brighter APB-3 puncta in *syd-2*, while there are fewer, less bright, and smaller UNC-101 puncta in *syd-2* animals. The effects of SYD-2 on APB-3 may be explained in two ways. AP-3 recruitment to membrane surfaces depends on binding to cargo proteins [82]. Therefore, after AP-3 has sorted cargo, SYD-2 may facilitate UNC-104 clustering, and thereby permit sufficient force generation to enable the exit of cargo proteins from an endosomal compartment. Multiple motors are known to generate greater pulling force and deformation of membrane compartments [83,84]. Moreover, the Kinesin 3 family motor KIF13A has been shown to physically bind the AP-1 complex to regulate trafficking of mannose-6-phosphate receptor and the melanosomal cargo, Tyrp1 [85,86]. SYD-2’s action may facilitate a similar role of UNC-104 in trafficking. An alternate possibility is that the kinetics of sorting is affected in the absence of SYD-2, leading to persistence of AP-3 complexes on membrane surfaces observed as an increase in the number of puncta in *syd-2* mutants. It is unclear how SYD-2 might influence the recruitment of the AP-1 complex to the Golgi. One possibility is that the changes in the AP-3 localization and potential changes in flux through the secretory pathway leads to slowing down of trafficking and consequent changes in the localization of AP-1 to reduce cargo jamming in Golgi and post-Golgi compartments.

Polarized trafficking of SVps, specifically their exclusion from dendrites, is dependent on both LRK-1 and the AP-3 complex. SNB-1 mistrafficking in both *lrk-1* and *apb-3* mutants is dependent on SYD-2 as well as the AP-1 complex (Fig 6B) [3,41]. The role of SYD-2 in preventing SNB-1 from entering the dendrite in *lrk-1* and *apb-3* mutants might be due to the reduced levels of AP-1 on the Golgi (Fig 6F–6J). Therefore, in the background of the *syd-*2(*ju37*) allele, which does not affect AP-1 localization, *lrk-1* and *apb-*3 mutants continue to mistraffic SNB-1 to dendrites (S7E–S7G Fig). The mistrafficking of SNB-1 into dendrites of *unc-101; syd-2* double mutants may be akin to the dendritic mislocalization of SVps in *unc-104* mutants [87]. Based on the localization data of the AP-1 complex, we believe that SYD-2 might act as a selective regulator of AP-1 for polarized distribution of SVp carriers. The phenotype in *unc-101; syd-2* might arise from a combination of both loss of polarized trafficking leading to mixing of cargo into carriers along with a lack of UNC-104 activation leading to dynein-dependent transport into the dendrite [87]. In *lrk-1* and *apb-3* mutants, SNB-1 gets missorted into vesicles targeted towards the dendrite via the AP-1 complex [3,41]. In the absence of SYD-2 in *lrk-1* and *apb-3* mutants, UNC-104 on the atypical SV protein carriers is not sufficiently activated/clustered therefore these carriers are dendritically targeted. Consistent with this, in the absence of UNC-104 SVps have been shown to enter dendrites [87,88]. However, in *syd-2* alone, there might be sufficient motor activity to prevent this dynein-dependent dendritic transport. Since the SV-lysosomes are only enriched in the region within the first 50 microns of the PLM neuron, the SV-lysosomes could also represent a mechanism to regulate the polarized sorting of proteins at the axon initial segment (AIS). Alternately, the AIS could act to trap the cargo at the AIS via mechanisms that have been reported to aid polarized cargo distribution in neurons [89–91].

In conclusion, we propose that in the SV biogenesis pathway, one key step is the sorting of SVps and lysosomal proteins via LRK-1 and the AP-3 complex. We also propose a role for the active zone protein SYD-2 as a regulator of SVp trafficking, acting downstream to the AP-3 complex and via UNC-104, and as a regulator of polarized distribution of SVps acting along with the AP-1 complex. We show that SYD-2 genetically interacts with and alters the localization of both the AP-3 and AP-1 complexes to regulate the transport and polarized distribution of SVp carriers in *C*. *elegans* neurons.

## Materials and methods

### Strain maintenance

*C*. *elegans* strains were grown and maintained at 20°C on NGM plates seeded with *E*. *coli* OP50 strain using standard methods [92]. BD Bacto-Petone and BD Agar for the NGM were sourced from Becton, Dickinson and Company NJ, USA. All Sigma salts and Sigma cholesterol were obtained from local distributors of Sigma and Merck products. L4 or 1-day adult animals were used for imaging in all cases. The strains used are listed in S1 Table. Some strains were provided by the CGC, which is funded by the NIH Office of Research Infrastructure Programs (P40 OD010440).

### Plasmid construction

Expression plasmids were generated using standard PCR-based subcloning techniques. The *mec-4*p::*ctns-1*::*mCherry* plasmid (TTpl 509) was generated by replacing the *unc-129*p from #KG371 [93] with *mec-4*p using *Hind*III and *Bam*HI restriction enzymes. The *str-3*p::*ctns-1*::*mCherry* was generated by replacing the *unc-129*p from #KG371 with *str-3*p using *Bam*HI and *Apa*I restriction sites. To generate the *mec-4*p::*sng-1*::*gfp* plasmid (TTpl 696), SNG-1::GFP was amplified from NM491 [94] and cloned into a *mec-4*p containing vector using *Nhe*I and *Eco*RV restriction sites. To generate *rab-3*p::*apb-3*::*gfp* (TTpl 796), APB-3 was amplified from genomic DNA using Phusion Polymerase and cloned into a *rab-3*p-containing vector using *Nhe*I and *Age*I restriction sites.

To generate touch neuron specific expression plasmids for *rab-*7 and *lmp-1* under the *mec-7* promoter [95], cloning was performed using the Gateway *in vitro* recombination system (Invitrogen, Carlsbad, CA) using Grant lab modified versions of MiniMos enabled vectors pCFJ1662 (Hygromycin resistant) and pCFJ910 (G418 resistant) (gifts from Erik Jorgensen, University of Utah, Addgene #51482): pCFJ1662 Pmec7 GTWY mNeonGreen let858 (34F6) or pCFJ1662 Pmec7 mNeonGreen GTWY let858 (34D4), and pCFJ910 Pmec7 mScarlet GTWY let858 (33B6). pDONR221 entry vectors containing coding regions for *lmp-1* and *rab-7* were recombined into neuronal destination vectors by Gateway LR clonase II reaction to generate C-/N- terminal fusions. Single-copy integrations were obtained by MiniMOS technology [96].

The SYD-2 deletion constructs were made by PCR amplifying the regions of SYD-2 surrounding the required deletion. PCR fragments were then joined by Gibson Assembly.

### Generation of transgenic *C*. *elegans*

Transgenic lines were generated by following standard microinjection procedure [97] using an Olympus Ix53 microscope equipped with 20× and 40× lenses, Narishige M-152 micromanipulator (Narishige, Japan), and Eppendorf Femtojet 2 microinjector (local distributors of Eppendorf products). The F2 progeny that inherited and stably expressed the extrachromosomal transgene were UV irradiated to generate integrated lines. Worms with 100% transmission were selected and outcrossed with the wildtype N2 strain five times. Detailed information on the concentration of plasmids and co-injection markers used is listed in S2 Table.

### Imaging

#### Static imaging

L4 or 1-day adult worms were immobilized using 30 mM sodium azide and mounted on 2–5% agarose pads. Images were acquired on an Olympus IX73 Epifluorescence microscope with an Andor EMCCD camera or the Olympus Fluoview FV1000 confocal laser scanning microscope or Olympus IX83 with Perkin Elmer Ultraview Spinning Disc confocal microscope fitted with a Hamamatsu EMCCD camera. Since AP-3 localization is sensitive to levels of ATP [73], static imaging of APB-3::GFP was performed using 5 mM Tetramisole. APB-3::GFP was imaged on Olympus Spin SR10 (SoRA, 50 μm disk) fitted with Teledyne Photometrics sCMOS camera.

#### Time-lapse imaging

L4 worms were anesthetized in 3 mM tetramisole (Sigma- Aldrich) and mounted on 5% agarose pads. Time-lapse images were acquired in Olympus IX83 with Perkin Elmer Ultraview Spinning Disc confocal microscope and a Hamamatsu EMCCD camera or the Olympus Fluoview FV1000 confocal laser scanning microscope. Dual color simultaneous imaging was performed at 3 frames per second (fps), dual color sequential imaging was done at 1.3 fps, and single fluorophore imaging for analysis of vesicle length was done at 5 fps. All movies were 3 minutes long, and the region of imaging in the PLM comprised the first 60–100 μm of the neuronal process immediately outside the cell body, with the cell body in the frame of imaging. Live imaging of EBP-2::GFP to assess microtubule polarity was carried out using an Olympus IX73 Epifluorescence microscope with an Andor EMCCD camera at 3 fps. UNC-104::GFP was imaged on Olympus Spin SR10 (SoRA, 50 μm disk) fitted with Teledyne Photometrics sCMOS camera.

### Analysis

All analysis was done using FIJI [98].

#### Co-migration analysis

Kymographs were generated from identical regions of the movie in both color channels utilizing the ImageJ plugin MultipleKymograph. The kymographs were then synchronized and the overlapping sloped lines were considered as co-migrating particles. Occasionally, the SNG-1 appears to decorate as small domains on a large moving CTNS-1 vesicle. For such instances, CTNS-1 and SNG-1 are considered to be co-transporting: (a) if their path of motion is identical, and (b) if the SNG-1 signal is contained within the CTNS-1 vesicle intensity. For dual-color co-migration analysis, number of moving vesicles were counted which were positive for GFP alone, mCherry alone, and vesicles positive for both GFP and mCherry. Total number of vesicles = number of vesicles positive only for GFP + number of vesicles only positive for RFP + number of vesicles positive for both GFP and RFP.

$\%\;co‐migrating\;vesicles=(\frac{Number\;of\;vesicles\;positive\;for\;both\;GFP\;and\;mCherry}{Total\;number\;of\;vesicles})\;x\;100$

Fraction of GFP-positive vesicles co-migrating with mCherry-positive vesicles = $\left(\frac{Number\;of\;vesicles\;positive\;for\;both\;GFP\;and\;mCherry}{Number\;of\;vesicles\;only\;positive\;for\;GFP+Number\;of\;vesicles\;positive\;for\;both\;GFP\;and\;mCherry}\right)$

For detailed methods, please refer to [99].

#### Quantitation of penetrance of CTNS-1 puncta that exit into PLM neurites

For each genotype, at least 30 animals were annotated to observe the extent of CTNS-1 (or RAB-7 or LMP-1) presence in the PLM major neurite. Penetrance was measured by calculating the number of animals in which CTNS-1 (or RAB-7 or LMP-1) was present at or beyond the first 25 μm and 50 μm away from the cell body.

#### Quantitating the direction of motion of CTNS-1-carrying compartments

Only moving CTNS-1-carrying compartments were analyzed for their direction of motion. For CTNS-1-marked compartments moving clearly in a particular direction, they were annotated as such. For those moving bidirectionally, their net displacement was used to identify their direction of motion. If the vesicle’s final position at the end of the kymograph was closer to the cell body than when it started, it was considered to have moved retrogradely. If the vesicle’s final position at the end of the kymograph was farther away from the cell body than when it started, it was considered to have moved anterogradely. For vesicles whose position at the end of the kymograph remained largely unchanged, they were either not considered for analysis or were assigned the direction in which they were moving immediately before the end of the kymograph, depending upon how discernible their direction of motion was.

#### Density of CTNS-1 in the ASI dendrite

The number of CTNS-1 puncta in the dendrite and the length of measurable region (ROI) in the dendrite from the cell body to the end was counted for each animal. The density of lysosomes per 10 μm was calculated as:

(Number of CTNS-1 puncta in the dendrite/Length of the dendrite ROI) × 10

#### Quantitation of intensity of UNC-101::GFP and APB-3::GFP puncta

For UNC-101::GFP, two regions were chosen–(i) the cell bodies of the head neurons and (ii) the cell bodies along the ventral nerve cord. For APB-3::GFP, neurons in three regions–the head, along the ventral cord, and the tail–were analyzed. For both UNC-101::GFP and APB-3::GFP, per cell body, the number of puncta was calculated on a plane with the best focus for that cell body. On the same plane, the size and intensity of each puncta were measured. A cytosolic region close to one of the puncta was chosen to measure puncta/cytosolic intensity. Puncta intensity was quantitated by dividing the intensity of each puncta by the cytosolic intensity. All the values of puncta intensity to cytosolic intensity per cell body were averaged and plotted.

#### Quantitation of intensity of moving UNC-104::GFP particles

Average intensity of individual moving trajectory was measured and subtracted by average intensity of the same trajectory three pixels above (corresponding to 1 second prior on a kymograph with time progression vertically downwards). Stationary regions of a trajectory corresponding to a pause were neglected from the analysis [53].

#### Flux and run length analysis

For flux calculations, all moving UNC-104::GFP traces were annotated on a kymograph. Number of anterogradely moving vesicles were counted and normalised to a 20 μm and 10 s region of the kymograph [53].

For analysis of run length, SNG-1::GFP-positive traces were annotated on kymographs as accurately as possible. Average segment run length of vesicles was calculated using an in-house macro written in Fiji, as described by Vasudevan et al., 2023 [100].

#### Vesicle length analysis

In every kymograph, random non-overlapping ROIs (regions of interest) were chosen to measure the size of the vesicles. These random ROIs were generated by (S1 Macro). Any macro-generated random ROI that overlapped with a previous ROI for that kymograph was not used for the analysis. Within each ROI, the length of each moving compartment was quantified by measuring the thickness of the sloped line along the x-axis. Such measurements were done at regions not overlapping with stationary particles or other moving particles.

#### Microtubule polarity

Kymographs were generated from live movies of EBP-2::GFP in the axonal and anterior dendritic regions of the PVD neuron imaged at 3 fps. The number of anterogradely and retrogradely moving EBP-2 were counted from the kymographs and plotted.

### Statistical analysis

All statistical analyses were performed using OriginLab 2019. Distributions were checked for normality using the Shapiro–Wilk test. Data that fit a normal distribution were compared using either one-way ANOVA with Tukey’s post-hoc test or Student’s t test. Data that did not fit a normal distribution were compared using the Mann–Whitney test. Differences were considered significant when the p-value < 0.05.

## Supporting information

S1 Fig (A) Schematic of the PLM neuron. The red box highlights the region of imaging in the proximal major neuronal process. The arrow indicates the direction of anterograde motion, away from the cell body into the neuronal process. (B) Kymographs from dual-color imaging of RAB-3 with MAN-II in WT, imaged simultaneously at 3 frames per second (fps). Green traces indicate moving RAB-3 vesicles. Scale bars x-axis: 5 μm, y-axis: 30 s. (C) Kymographs from dual-color imaging of SNB-1 with CTNS-1 in WT, imaged sequentially at 1.3 fps. Green traces indicate moving SNB-1 vesicles, yellow traces indicate moving vesicles co-transporting SNB-1 and CTNS-1, and red traces indicate moving CTNS-1 vesicles. Scale bars x-axis: 5 μm, y-axis: 30 s. (D) Kymographs from dual-color imaging of RAB-3 with CTNS-1, imaged simultaneously at 3 fps. Green traces indicate moving RAB-3 vesicles, yellow traces indicate moving vesicles co-transporting RAB-3 and CTNS-1, and red traces indicate moving CTNS-1 vesicles. Scale bars x-axis: 5 μm, y-axis: 10 s. (E) Kymographs from dual-color imaging of mNeonGreen::RAB-7 with CTNS-1::mCherry, imaged sequentially at 1.3 fps. Green traces indicate moving RAB-7 vesicles, yellow traces indicate moving vesicles co-transporting RAB-7 and CTNS-1, and red traces indicate moving CTNS-1 vesicles. Scale bars x-axis: 5 μm, y-axis: 30 s. (F) Kymographs from dual-color imaging of SNB-1 with RAB-3, imaged simultaneously at 3 fps. Green traces indicate moving SNB-1 vesicles, yellow traces indicate moving vesicles co-transporting SNB-1 and RAB-3, and red traces indicate moving RAB-3 vesicles. Scale bars x-axis: 5 μm, y-axis: 10 s. (G) Kymographs from dual-color imaging of SNG-1 with RAB-3, imaged sequentially at 1.3 fps. Green traces indicate moving SNG-1 vesicles, yellow traces indicate moving vesicles co-transporting SNG-1 and RAB-3, and red traces indicate moving RAB-3 vesicles. Scale bars x-axis: 5 μm, y-axis: 30 s. (H) Penetrance for the number of animals in which CTNS-1 localizes up to 25 μm of the PLM neuronal process away from the cell body. Numbers inside the bars indicate the number of animals per genotype. Numbers above the bars indicate the penetrance values. For bar graphs with very little height, the lower number indicates the number of animals for that genotype while the number above indicates the penetrance value. (I) Penetrance for the number of animals in which RAB-7 localizes up to 25 μm of the PLM neuronal process away from the cell body. Numbers inside the bars indicate the number of animals per genotype. Numbers above the bars indicate the penetrance values. (J) Penetrance for the number of animals in which LMP-1 localizes up to 25 μm of the PLM neuronal process away from the cell body. Numbers inside the bars indicate the number of animals per genotype. Numbers above the bars indicate the penetrance values. For bar graphs with very little height, the lower number indicates the number of animals for that genotype while the number above indicates the penetrance value. (K) Penetrance for the number of animals in which CTNS-1 localizes up to 50 μm of the PLM neuronal process away from the cell body in WT and *syd-2*(*ok217*). Numbers inside the bars indicate the number of animals per genotype. Numbers above the bars indicate the penetrance values.
(TIF)

S2 Fig (A) Venn Diagrams depicting the fraction of moving vesicles transporting a SV protein, a lysosomal protein or both. SV proteins are in shades of red while late endosomal/lysosomal proteins are in shades of blue. Sizes of sets are representative of the fraction of moving vesicles positive for that marker. (B) Velocities of moving CTNS-1 compartments when expressed alone compared to velocities of moving CTNS-1 compartments co-transporting various SV proteins. Graphs on the left measure velocities of anterogradely moving CTNS-1 compartments, while those on the right are retrogradely moving CTNS-1 compartments, measured in μm/s. Statistical significance in orange text is for anterogradely moving vesicles, while that in black is for the retrogradely moving vesicles. Number of animals, N = 9; All comparisons to respective values from CTNS-1 alone compartments. P-values > 0.05 (Kruskal-Wallis ANOVA with Dunn’s posthoc test); ns: not significant. # P-values ≤ 0.05 (Kruskal-Wallis ANOVA with Dunn’s posthoc test). (C) Velocity of moving CTNS-1 compartments with and without SNG-1 measured in μm/s in animals expressing transgenes for both CTNS-1 and SNG-1. Number of animals, N = 16; number of vesicles, n = 63; P-value > 0.05 (Mann–Whitney Test); ns: not significant. (D) Lengths of moving CTNS-1 compartments with and without SNG-1. # P-value ≤ 0.05 (Mann-Whitney test). Number of animals, N = 16; number of vesicles, n = 6.
(TIF)

S3 Fig (A) Kymographs from sequential dual-color imaging of SNG-1 and CTNS-1 at 1.3 fps in WT, *lrk-1*(*km17*), and *apb-3*(*ok429*). Green traces indicate moving SNG-1-carrying vesicles, yellow traces indicate moving vesicles co-transporting SNG-1 and CTNS-1, and red traces indicate moving CTNS-1-carrying vesicles. Scale bar x-axis: 5 μm and y-axis: 30 s. (B) Kymographs from sequential dual-color imaging of SNG-1 and RAB-7 at 1.3 fps in WT, *lrk-1*(*km17*), and *apb-3*(*ok429*). Green traces indicate moving SNG-1-carrying vesicles, yellow traces indicate moving vesicles co-transporting SNG-1 and RAB-7, and red traces indicate moving RAB-7-carrying vesicles. Scale bar x-axis: 5 μm and y-axis: 30 s. (C) Quantitation of percentage of total moving vesicles transporting SNG-1 or CTNS-1 or both from kymograph analysis of dual color imaging. (D) Quantitation of percentage of total moving vesicles transporting SNG-1 or RAB-7 or both from kymograph analysis of dual color imaging. (E) Quantitation of fraction of total moving CTNS-1-carrying vesicles co-transporting SNG-1 from kymograph analysis of dual color imaging. ^#^P-values ≤ 0.05 **(**Mann–Whitney Test, all comparisons to WT); ns: not significant; Number of animals per genotype (N) ≥ 20; Number of vesicles (n) > 400. (F) Quantitation of fraction of total moving RAB-7-carrying vesicles co-transporting SNG-1 from kymograph analysis of dual color imaging. # P-values ≤ 0.05 **(**Mann–Whitney Test, all comparisons to WT); ns: not significant; N ≥ 20 per genotype; n > 400.
(TIF)

S4 Fig (A) Quantitation of fraction of total moving SNB-1-carrying vesicles co-transporting CTNS-1 in WT, *lrk-1*(*km17*), and *apb-3*(*ok429*) from kymograph analysis of dual color imaging. P-values > 0.05 **(**Mann-Whitney test, all comparisons to WT); ns: not significant; N ≥ 15 per genotype; n > 400. (B) Quantitation of fraction of total moving RAB-3-carrying vesicles co-transporting CTNS-1 in WT, *lrk-1*(*km17*), and *apb-3*(*ok429*) from kymograph analysis of simultaneous dual color imaging at 3 fps. # P-values ≤ 0.05 **(**Mann–Whitney Test, all comparisons to WT); ns: not significant; N = 5 per genotype; n > 500. (C) Quantitation of percentage of total moving vesicles transporting RAB-3, CTNS-1 or both in WT, *lrk-1*(*km17*), and *apb-3*(*ok429*) from kymograph analysis of simultaneous dual color imaging at 3 fps. (D) Length of moving CTNS-1-carrying vesicles in WT, *lrk*-*1* and *apb-3*. # P-values ≤ 0.05 **(**Mann–Whitney Test, all comparisons to WT); ns: not significant. Number of animals (N) > 49 per genotype; number of vesicles, n > 500. (E) Percentages of cell bodies of WT, *lrk-1*, and *syd-2* with APB-3::GFP puncta. N > 10 per genotype; n > 75 cell bodies. (F) GFP::RAB-3 in the cell body, process, and synapses of PLM neurons shows dependence on UNC-104 in *lrk-1*(*km17*), *apb-3*(*ok429*), and *syd-2*(*ok217*) mutants, and their doubles with *unc-104*(*e1265tb120*). Red arrow point to RAB-3::GFP signal at PLM synapses. Scale bar: 10 μm.
(TIF)

S5 Fig (A) Quantitation of the number of CTNS-1-labeled compartments per 10 μm of the PLM major neurite proximal to the cell body in WT, *unc-104*(*e1265tb120*), and *syd-2*(*ok217*). # P-values ≤ 0.05 **(**Mann–Whitney Test, all comparisons to WT); ns: not significant; Number of animals (N) ≥ 20 per genotype; Number of CTNS-1-labeled compartments (n) ≥ 70. (B) Quantitation of fraction of total moving SNB-1-carrying vesicles co-transporting CTNS-1 in WT and *syd-2*(*ok217*), from kymograph analysis of sequential dual color imaging at 1.3 fps. P-value > 0.05 (Mann–Whitney Test); ns: not significant; N > 15 per genotype; n > 750 vesicles. (C) Quantitation of fraction of total moving RAB-3-carrying vesicles co-transporting CTNS-1, in WT and *syd-2*(*ok217*), from kymograph analysis of simultaneous dual color imaging at 3 fps. P-value > 0.05 (Mann–Whitney Test); ns: not significant; N = 5; n > 500 vesicles. (D) Schematic of *C*. *elegans* SYD-2 protein with all domains labelled and the various *syd-2* alleles highlighted. The dotted lines represent the Intrinsically Disordered Regions (IDR) of SYD-2 that are essential for lipid-lipid phase separation (LLPS). (E) Schematics representing the various SYD-2 deletion constructs. Dotted lines represent the deleted regions of SYD-2. (F) CTNS-1::mCherry in cell body and process of PLM neurons in WT, *syd-2* and *apb-3*; *syd-2* mutants that express the various SYD-2 constructs. Red arrows point to CTNS-1::mCherry signal in the PLM neuronal process. Scale bar: 10 μm. (G) Penetrance for the number of animals in which CTNS-1 is seen beyond 50 μm of the PLM neuronal process away from the cell body in WT, *syd-2* and *apb-3*; *syd-2* mutants that express the various SYD-2 constructs.
(TIF)

S6 Fig (A) Penetrance for the number of animals in which SNG-1 is seen beyond 50 μm of the PLM neuronal process away from the cell body in *unc-104(e1265)*, *syd-2(ok217)* and *unc-104; syd-2* double mutants. Numbers inside the bars indicate the number of animals per genotype. Numbers above the bars indicate the penetrance values. For very short bar graphs, the lower number indicates the number of animals for that genotype while the number above indicates the penetrance value. (B) Penetrance for the number of animals in which SNG-1 is seen beyond 150 μm of the PLM neuronal process away from the cell body in *unc-104(e1265)*, *syd-2(ok217)* alone and in various mutant combinations with *lrk-1(km17)* and *apb-3(ok429)*. Numbers inside the bars indicate the number of animals per genotype. Numbers above the bars indicate the penetrance values. For very short bar graphs, the lower number indicates the number of animals for that genotype while the number above indicates the penetrance value. (C) Penetrance for the number of animals in which SNB-1 is seen beyond 50 μm of the PLM neuronal process away from the cell body in *unc-104(e1265)*, *syd-2(ok217)* and *unc-104; syd-*2 double mutants. Numbers inside the bars indicate the number of animals per genotype. Numbers above the bars indicate the penetrance values. For very short bar graphs, the lower number indicates the number of animals for that genotype while the number above indicates the penetrance value. (D) Penetrance for the number of animals in which SNB-1 is seen beyond 150 μm of the PLM neuronal process away from the cell body in *unc-104(e1265)*, *syd-2(ok217)* alone and in various mutant combinations with *lrk-1(km17)* and *apb-3(ok429)*. Numbers inside the bars indicate the number of animals per genotype. Numbers above the bars indicate the penetrance values. For very short bar graphs, the lower number indicates the number of animals for that genotype while the number above indicates the penetrance value. (E) Schematic of PLM neuron. Red box highlights the region of PLM where UNC-104::GFP was imaged. (F) Kymographs of UNC-104::GFP movies imaged at 3 frames per second (fps), showing moving UNC-104:GFP trajectories in PLM neuronal process. Scale bar x-axis: 5 μm and y-axis: 10 s. (G) Background subtracted intensity of moving UNC-104::GFP trajectories in WT and *syd-2*(*ok217*). Number of animals per genotype (N) > 10; number of vesicles per genotype (n) > 1500. P-value > 0.05 (Mann–Whitney Test); ns: not significant. (H) Number of anterogradely moving UNC-104::GFP trajectories per 20 μm per 10 seconds in WT and *syd-2*(*ok217*). P-value 0.05 (One-Way ANOVA with Tukey’s post-hoc test); ns: not significant. (I) Average displacement length of anterogradely moving SNG-1::GFP trajectory in WT and *syd-2*(*ok217*) in μm. # P-values ≤ 0.05 **(**two tailed Student’s T test); N > 8 animals per genotype. (J) Average displacement length of retrogradely moving SNG-1::GFP trajectory in WT and *syd-2*(*ok217*) in μm. P-values > 0.05 **(**two tailed Student’s T test); ns: not significant; N > 8 animals per genotype.
(TIF)

S7 Fig (A) Quantitation of fraction of EBP-2::GFP comets moving in either anterograde or retrograde directions in both the axon and the anterior dendrite of WT and *syd-2*(*ok217*); Number of animals (N) > 8 for each genotype; Number of comets analyzed (n) > 150. (B) Schematic of the AWC neuron with a red box highlighting the region of imaging. ODR-1::GFP in the dendrite and axon of the AWC neuron. Red arrow points to the ODR-1::GFP signal in the AWC axon in *syd-2*(*ok217*), *apb-3*(*ok429*), and *unc-101*(*m1*). Scale bar: 20 μm. (C) Quantitation of sizes of moving RAB-3 containing SVp carriers in WT, *syd-2*(*ok217*), *unc-101*(*m1*), and *unc-101*; *syd-2*. The x-axis depicts the length (in μm) of moving RAB-3 carrying SVp carriers. The y-axis depicts the percentage of moving RAB-3 carrying SVp carriers of various lengths. Number of animals (N) ≥ 9 per genotype; Number of vesicles (n) > 400. (D) Quantitation of the number of UNC-101::GFP puncta per cell body in WT and *syd-2*(*ok217*). P-value > 0.05 (Mann–Whitney Test); N > 5 animals; n > 25 cell bodies. (E) Images show UNC-101::GFP puncta in the cell bodies of the ventral nerve cord neurons in WT and *syd-2*(*ju37*). Scale bar: 10 μm. (F) Quantitation of the average size of UNC-101::GFP puncta per cell body in WT and *syd-2*(*ju37*). P-value > 0.05 (Mann–Whitney Test); ns: not significant; N > 5 animals; n > 25 cell bodies. (G) Quantitation of intensity of UNC-101::GFP puncta in the cell bodies of the ventral nerve cord in WT and *syd-2*(*ju37*). The ratio of the intensity of UNC-101::GFP puncta to cytosolic intensity in the cell body is plotted. P-value > 0.05 (Mann–Whitney test); ns: not significant; N > 5 animals; n > 10 cell bodies.
(TIF)

S1 Macro Macro for generating random ROIs. (IJM)

S1 Table List of strains. (XLSX)

S2 Table List of plasmids used for strain generation. (XLSX)

S1 Movie CTNS-1 and SNG-1 in WT. SNG-1::GFP and CTNS-1::mCherry in the PLM neuronal process. Imaged sequentially at 1.3 frames per second (fps), playback at 20 fps. Genotype: wildtype. Cell body on the right.
(AVI)

S2 Movie RAB-7 and SNG-1 in WT. SNG-1::GFP and mScarlet::RAB-7 in the PLM neuronal process. Imaged sequentially at 1.3 frames per second (fps), playback at 20 fps. Genotype: wildtype. Cell body on the right.
(AVI)

S3 Movie CTNS-1 and SNG-1 in *lrk-1*. SNG-1::GFP and CTNS-1::mCherry in the PLM neuronal process. Imaged sequentially at 1.3 frames per second (fps), playback at 20 fps. Genotype: *lrk-1*(*km17*). Cell body on the right.
(AVI)

S4 Movie CTNS-1 and SNG-1 in *apb-3*. SNG-1::GFP and CTNS-1::mCherry in the PLM neuronal process. Imaged sequentially at 1.3 frames per second (fps), playback at 20 fps. Genotype: *apb-3*(*ok429*). Cell body on the right.
(AVI)

S5 Movie RAB-7 and SNG-1 in *lrk-1*. SNG-1::GFP and mScarlet::RAB-7 in the PLM neuronal process. Imaged sequentially at 1.3 frames per second (fps), playback at 20 fps. Genotype: *lrk-1*(*km17*). Cell body on the right.
(AVI)

S6 Movie CTNS-1 and SNG-1 in *unc-104*. SNG-1::GFP and CTNS-1::mCherry in the PLM neuronal process. Imaged sequentially at 1.3 frames per second (fps), playback at 20 fps. Genotype: *unc-104*(*e1265tb120*). Cell body on the right.
(AVI)

S7 Movie CTNS-1 and SNG-1 in *syd-2*. SNG-1::GFP and CTNS-1::mCherry in the PLM neuronal process. Imaged sequentially at 1.3 frames per second (fps), playback at 20 fps. Genotype: *syd-2*(*ok217*). Cell body on the right.
(AVI)

S1 Text **Table A. Statistics for fraction of SNG-1 co-migrating with RAB-3. Associated with Figs 2A and 4G. Table B. Statistics for fraction of SNG-1 co-migrating with CTNS-1. Associated with Figs 2B and 4A. Table C. Statistics for fraction of CTNS-1 co-migrating with SNG-1. Associated with S3E Fig. Table D. Statistics for fraction of SNG-1 co-migrating with RAB-7. Associated with Figs 2C and 4B. Table E. Statistics for fraction of RAB-7 co-migrating with SNG-1. Associated with S3F Fig. Table F. Statistics for fraction of SNB-1 co-migrating with CTNS-1. Associated with S4A and S5B Figs. Table G. Statistics for fraction of RAB-3 co-migrating with CTNS-1. Associated with S4B and S5C Figs. Table H. Analysis of APB-3::GFP puncta number per cell body, size and intensity. Associated with Fig 2H–2J. Table I. Statistics for percentage of vesicles with co-migrating SNB-1 and RAB-3. Associated with Fig 4F. Table J. Analysis of UNC-101::GFP puncta number per cell body, size, and intensity in neurons of head. Associated with Figs 6G, 6H and** S7D**. Table K. Analysis of UNC-101::GFP puncta number per cell body, size, and intensity in neurons of ventral nerve cord–WT vs. *syd-2*(*ok217*). Associated with Fig 6J. Table L. Analysis of UNC-101::GFP puncta number per cell body, size, and intensity in neurons of ventral nerve cord–WT vs. *syd-2*(*ju37*). Associated with S7F and S7G Fig. Table M. Analysis of intensity of moving UNC-104::GFP. Associated with S6G Fig. Table N. Analysis of number of anterogradely moving UNC-104::GFP particles per 20 μm per 10 seconds. Associated with S6H Fig. Table O. Analysis of average run length of anterogradely moving SNG-1-carrying vesicles (μm). Associated with S6I Fig. Table P. Analysis of average run length of retrogradely moving SNG-1-carrying vesicles (μm). Associated with S6J Fig. Table Q. Analysis of velocities of CTNS-1-positive compartments moving with and without other markers. Associated with S2B Fig. Table R. Analysis of lengths of CTNS-1-positive compartments with and without SNG-1. Associated with S2C Fig. Table S. Analysis of velocities of CTNS-1-positive compartments with and without SNG-1. Associated with S2D Fig. Table T. Summary of all phenotypes.**
(XLSX)
