# Supplementary figures and images for: LRK-1/LRRK2 and AP-3 regulate trafficking of synaptic vesicle precursors through active zone protein SYD-2/Liprin-α
Source: PLoS Genet. 2024 May 9;20(5):e1011253. doi: 10.1371/journal.pgen.1011253 (PMC11081264; doi:10.1371/journal.pgen.1011253)

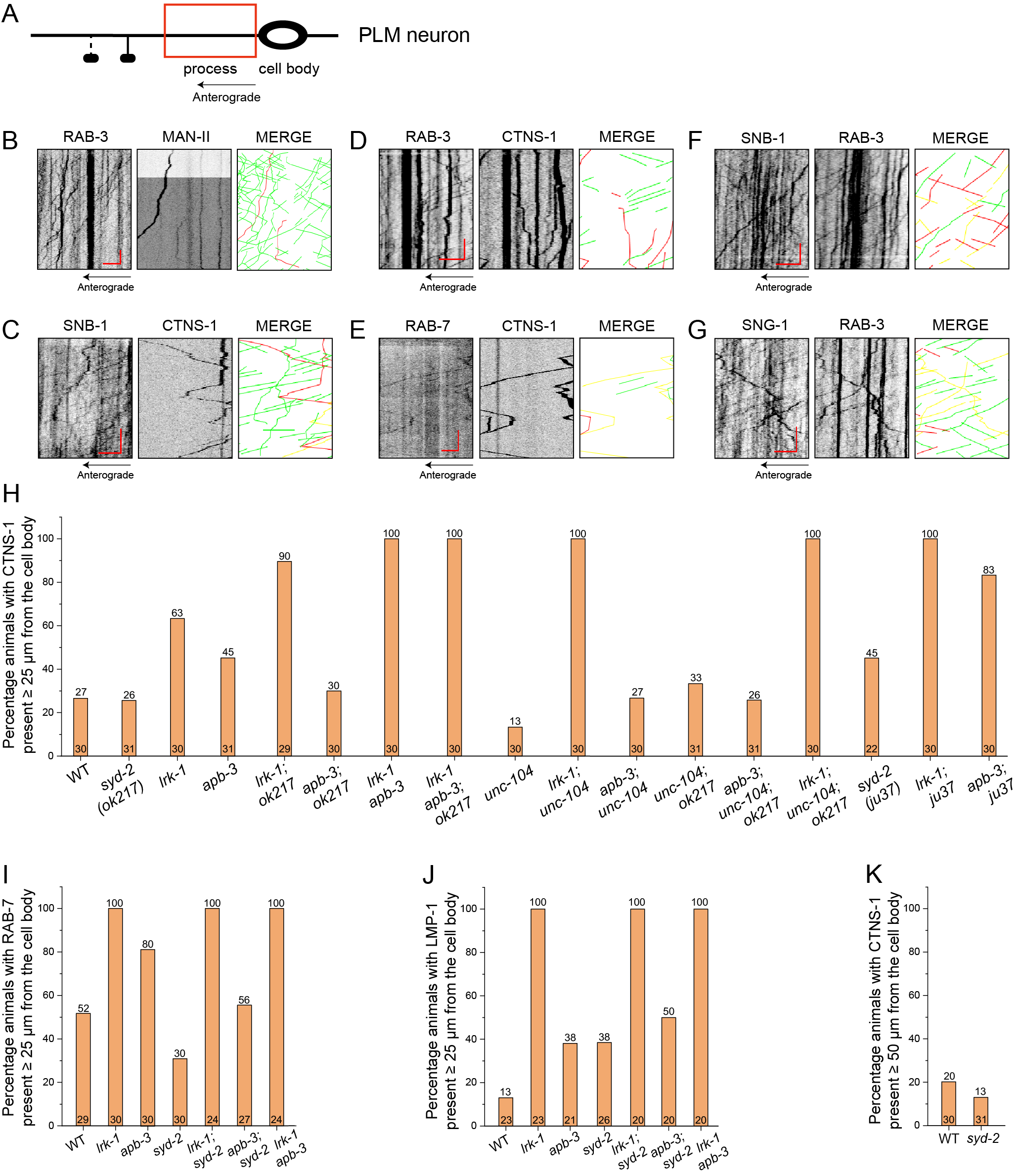

Supplement: S1 Fig — (A) Schematic of the PLM neuron. The red box highlights the region of imaging in the proximal major neuronal process. The arrow indicates the direction of anterograde motion, away from the cell body into the neuronal process. (B) Kymographs from dual-color imaging of RAB-3 with MAN-II in WT, imaged simultaneously at 3 frames per second (fps). Green traces indicate moving RAB-3 vesicles. Scale bars x-axis: 5 μm, y-axis: 30 s. (C) Kymographs from dual-color imaging of SNB-1 with CTNS-1 in WT, imaged sequentially at 1.3 fps. Green traces indicate moving SNB-1 vesicles, yellow traces indicate moving vesicles co-transporting SNB-1 and CTNS-1, and red traces indicate moving CTNS-1 vesicles. Scale bars x-axis: 5 μm, y-axis: 30 s. (D) Kymographs from dual-color imaging of RAB-3 with CTNS-1, imaged simultaneously at 3 fps. Green traces indicate moving RAB-3 vesicles, yellow traces indicate moving vesicles co-transporting RAB-3 and CTNS-1, and red traces indicate moving CTNS-1 vesicles. Scale bars x-axis: 5 μm, y-axis: 10 s. (E) Kymographs from dual-color imaging of mNeonGreen::RAB-7 with CTNS-1::mCherry, imaged sequentially at 1.3 fps. Green traces indicate moving RAB-7 vesicles, yellow traces indicate moving vesicles co-transporting RAB-7 and CTNS-1, and red traces indicate moving CTNS-1 vesicles. Scale bars x-axis: 5 μm, y-axis: 30 s. (F) Kymographs from dual-color imaging of SNB-1 with RAB-3, imaged simultaneously at 3 fps. Green traces indicate moving SNB-1 vesicles, yellow traces indicate moving vesicles co-transporting SNB-1 and RAB-3, and red traces indicate moving RAB-3 vesicles. Scale bars x-axis: 5 μm, y-axis: 10 s. (G) Kymographs from dual-color imaging of SNG-1 with RAB-3, imaged sequentially at 1.3 fps. Green traces indicate moving SNG-1 vesicles, yellow traces indicate moving vesicles co-transporting SNG-1 and RAB-3, and red traces indicate moving RAB-3 vesicles. Scale bars x-axis: 5 μm, y-axis: 30 s. (H) Penetrance for the number of animals in which CTNS-1 loc [file pgen.1011253.s001.tif]

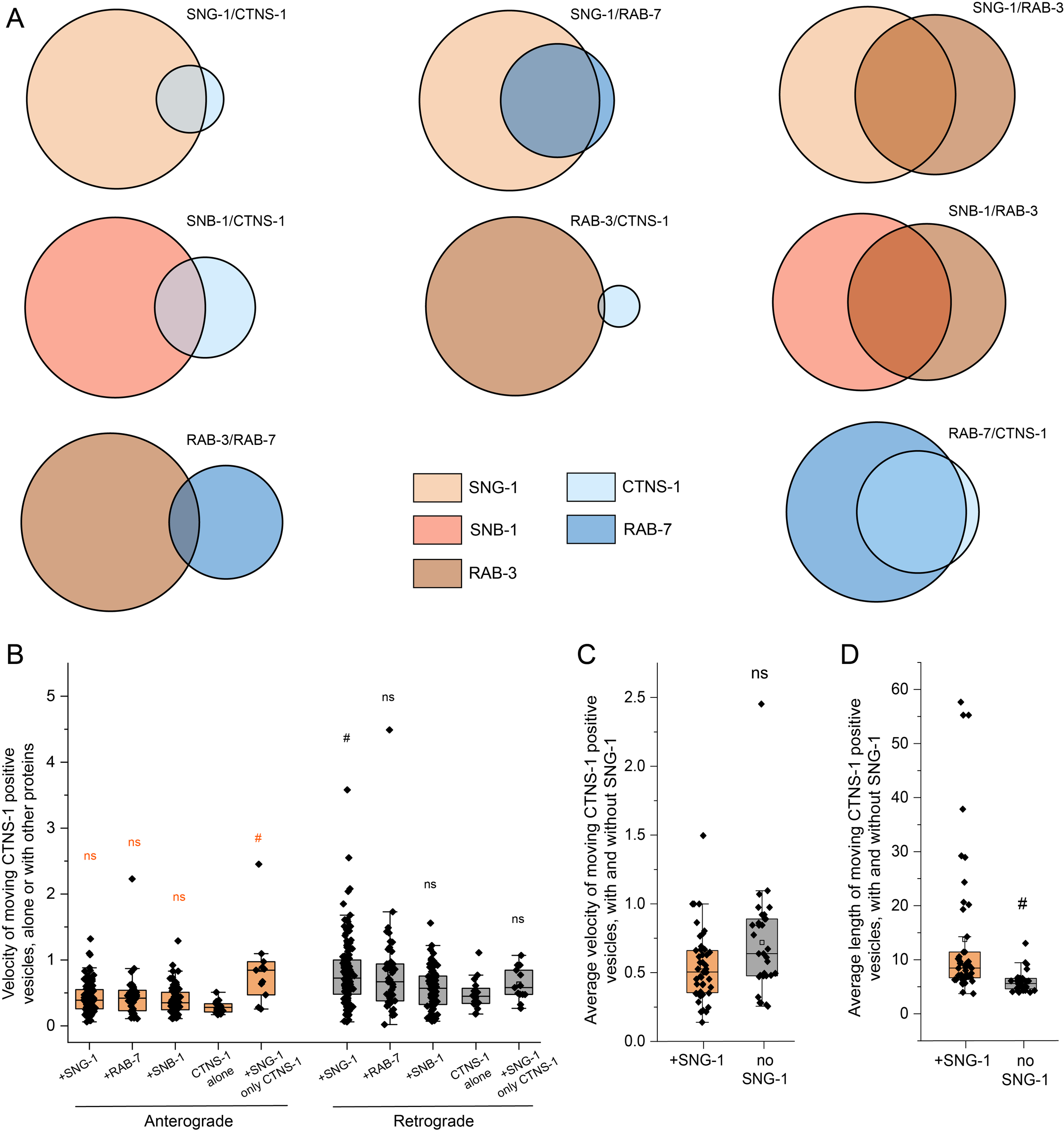

Supplement: S2 Fig — (A) Venn Diagrams depicting the fraction of moving vesicles transporting a SV protein, a lysosomal protein or both. SV proteins are in shades of red while late endosomal/lysosomal proteins are in shades of blue. Sizes of sets are representative of the fraction of moving vesicles positive for that marker. (B) Velocities of moving CTNS-1 compartments when expressed alone compared to velocities of moving CTNS-1 compartments co-transporting various SV proteins. Graphs on the left measure velocities of anterogradely moving CTNS-1 compartments, while those on the right are retrogradely moving CTNS-1 compartments, measured in μm/s. Statistical significance in orange text is for anterogradely moving vesicles, while that in black is for the retrogradely moving vesicles. Number of animals, N = 9; All comparisons to respective values from CTNS-1 alone compartments. P-values > 0.05 (Kruskal-Wallis ANOVA with Dunn’s posthoc test); ns: not significant. # P-values ≤ 0.05 (Kruskal-Wallis ANOVA with Dunn’s posthoc test). (C) Velocity of moving CTNS-1 compartments with and without SNG-1 measured in μm/s in animals expressing transgenes for both CTNS-1 and SNG-1. Number of animals, N = 16; number of vesicles, n = 63; P-value > 0.05 (Mann–Whitney Test); ns: not significant. (D) Lengths of moving CTNS-1 compartments with and without SNG-1. # P-value ≤ 0.05 (Mann-Whitney test). Number of animals, N = 16; number of vesicles, n = 6. (TIF) [file pgen.1011253.s002.tif]

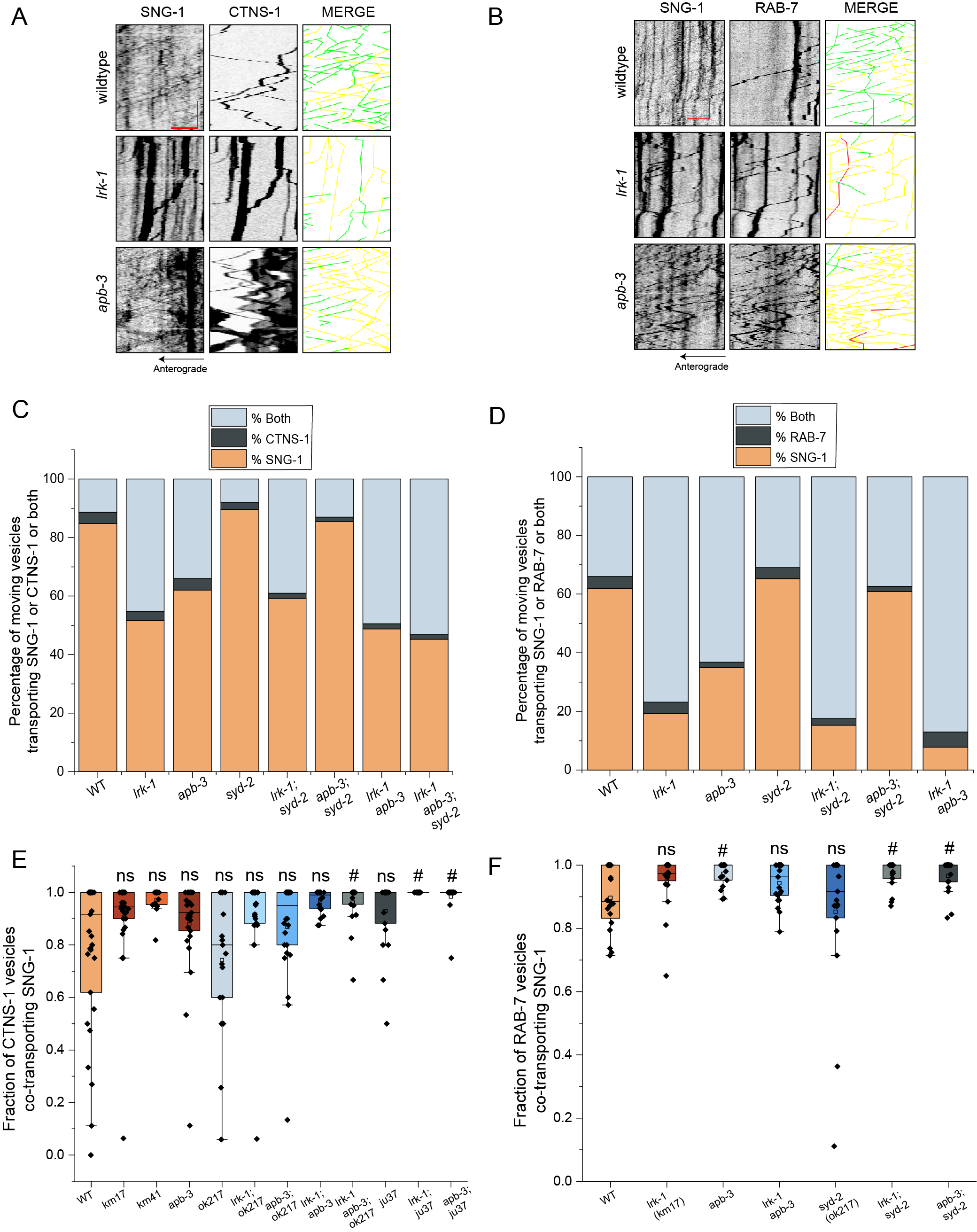

Supplement: S3 Fig — (A) Kymographs from sequential dual-color imaging of SNG-1 and CTNS-1 at 1.3 fps in WT, lrk-1(km17), and apb-3(ok429). Green traces indicate moving SNG-1-carrying vesicles, yellow traces indicate moving vesicles co-transporting SNG-1 and CTNS-1, and red traces indicate moving CTNS-1-carrying vesicles. Scale bar x-axis: 5 μm and y-axis: 30 s. (B) Kymographs from sequential dual-color imaging of SNG-1 and RAB-7 at 1.3 fps in WT, lrk-1(km17), and apb-3(ok429). Green traces indicate moving SNG-1-carrying vesicles, yellow traces indicate moving vesicles co-transporting SNG-1 and RAB-7, and red traces indicate moving RAB-7-carrying vesicles. Scale bar x-axis: 5 μm and y-axis: 30 s. (C) Quantitation of percentage of total moving vesicles transporting SNG-1 or CTNS-1 or both from kymograph analysis of dual color imaging. (D) Quantitation of percentage of total moving vesicles transporting SNG-1 or RAB-7 or both from kymograph analysis of dual color imaging. (E) Quantitation of fraction of total moving CTNS-1-carrying vesicles co-transporting SNG-1 from kymograph analysis of dual color imaging. #P-values ≤ 0.05 (Mann–Whitney Test, all comparisons to WT); ns: not significant; Number of animals per genotype (N) ≥ 20; Number of vesicles (n) > 400. (F) Quantitation of fraction of total moving RAB-7-carrying vesicles co-transporting SNG-1 from kymograph analysis of dual color imaging. # P-values ≤ 0.05 (Mann–Whitney Test, all comparisons to WT); ns: not significant; N ≥ 20 per genotype; n > 400. (TIF) [file pgen.1011253.s003.tif]

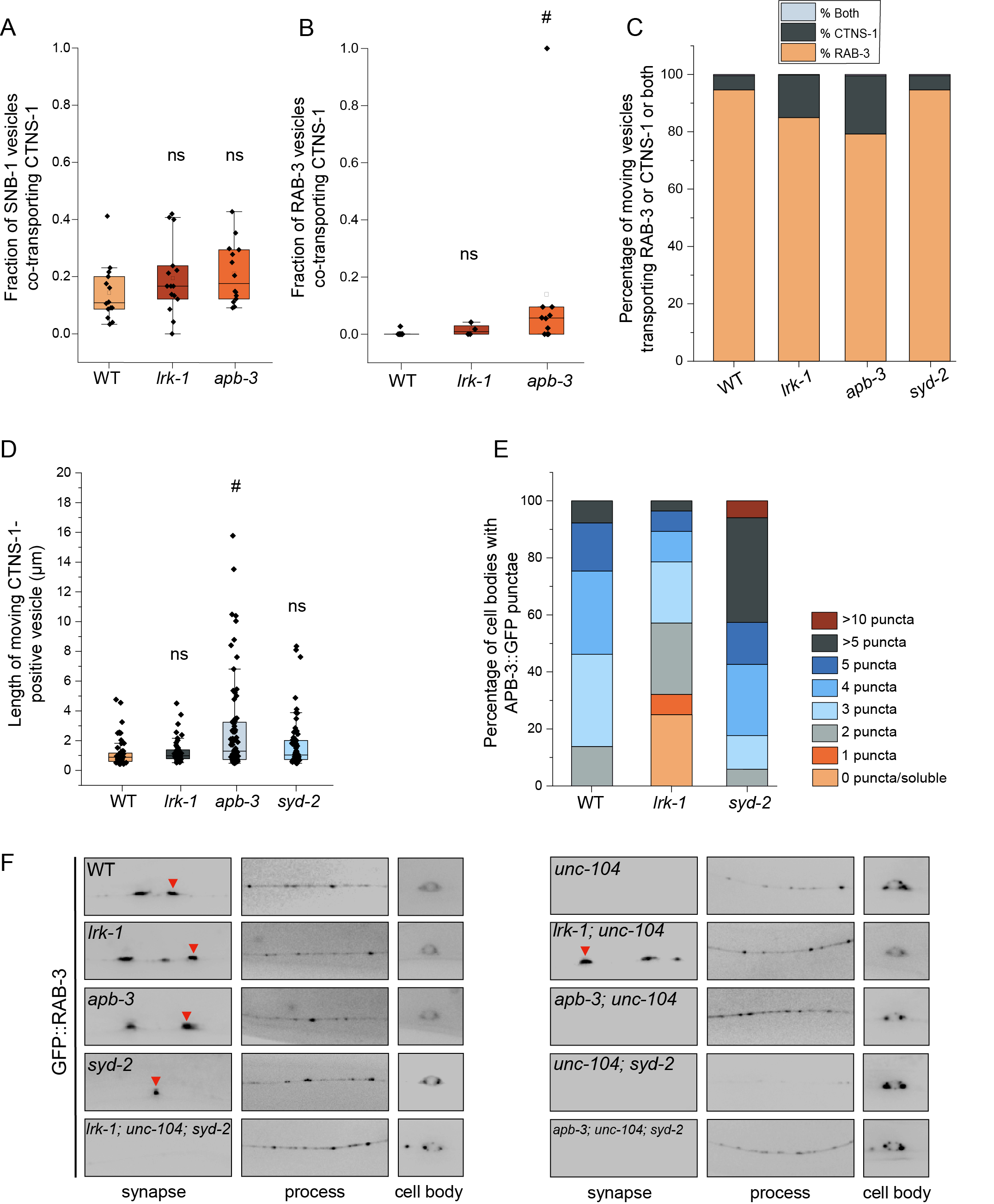

Supplement: S4 Fig — (A) Quantitation of fraction of total moving SNB-1-carrying vesicles co-transporting CTNS-1 in WT, lrk-1(km17), and apb-3(ok429) from kymograph analysis of dual color imaging. P-values > 0.05 (Mann-Whitney test, all comparisons to WT); ns: not significant; N ≥ 15 per genotype; n > 400. (B) Quantitation of fraction of total moving RAB-3-carrying vesicles co-transporting CTNS-1 in WT, lrk-1(km17), and apb-3(ok429) from kymograph analysis of simultaneous dual color imaging at 3 fps. # P-values ≤ 0.05 (Mann–Whitney Test, all comparisons to WT); ns: not significant; N = 5 per genotype; n > 500. (C) Quantitation of percentage of total moving vesicles transporting RAB-3, CTNS-1 or both in WT, lrk-1(km17), and apb-3(ok429) from kymograph analysis of simultaneous dual color imaging at 3 fps. (D) Length of moving CTNS-1-carrying vesicles in WT, lrk-1 and apb-3. # P-values ≤ 0.05 (Mann–Whitney Test, all comparisons to WT); ns: not significant. Number of animals (N) > 49 per genotype; number of vesicles, n > 500. (E) Percentages of cell bodies of WT, lrk-1, and syd-2 with APB-3::GFP puncta. N > 10 per genotype; n > 75 cell bodies. (F) GFP::RAB-3 in the cell body, process, and synapses of PLM neurons shows dependence on UNC-104 in lrk-1(km17), apb-3(ok429), and syd-2(ok217) mutants, and their doubles with unc-104(e1265tb120). Red arrow point to RAB-3::GFP signal at PLM synapses. Scale bar: 10 μm. (TIF) [file pgen.1011253.s004.tif]

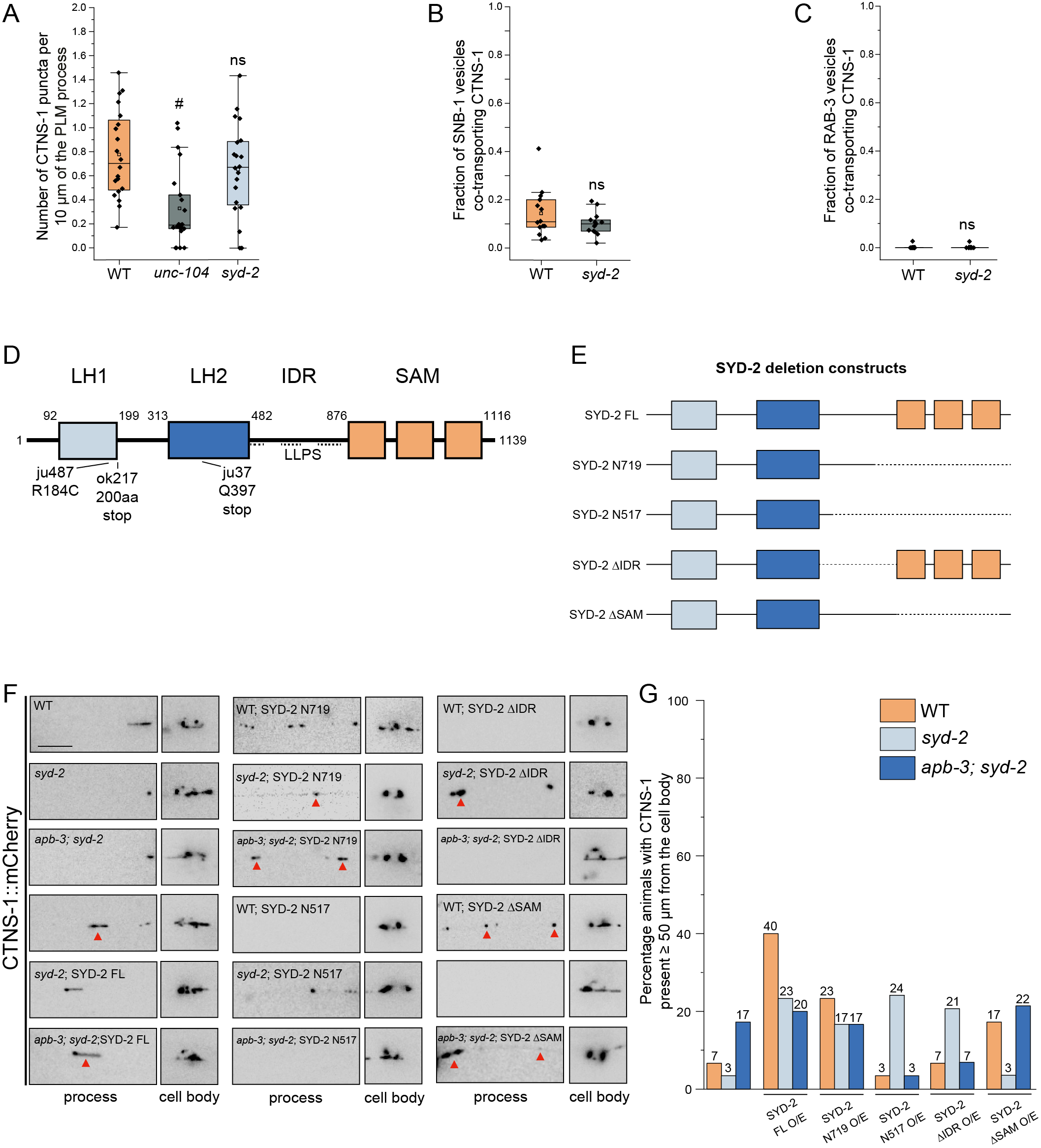

Supplement: S5 Fig — (A) Quantitation of the number of CTNS-1-labeled compartments per 10 μm of the PLM major neurite proximal to the cell body in WT, unc-104(e1265tb120), and syd-2(ok217). # P-values ≤ 0.05 (Mann–Whitney Test, all comparisons to WT); ns: not significant; Number of animals (N) ≥ 20 per genotype; Number of CTNS-1-labeled compartments (n) ≥ 70. (B) Quantitation of fraction of total moving SNB-1-carrying vesicles co-transporting CTNS-1 in WT and syd-2(ok217), from kymograph analysis of sequential dual color imaging at 1.3 fps. P-value > 0.05 (Mann–Whitney Test); ns: not significant; N > 15 per genotype; n > 750 vesicles. (C) Quantitation of fraction of total moving RAB-3-carrying vesicles co-transporting CTNS-1, in WT and syd-2(ok217), from kymograph analysis of simultaneous dual color imaging at 3 fps. P-value > 0.05 (Mann–Whitney Test); ns: not significant; N = 5; n > 500 vesicles. (D) Schematic of C. elegans SYD-2 protein with all domains labelled and the various syd-2 alleles highlighted. The dotted lines represent the Intrinsically Disordered Regions (IDR) of SYD-2 that are essential for lipid-lipid phase separation (LLPS). (E) Schematics representing the various SYD-2 deletion constructs. Dotted lines represent the deleted regions of SYD-2. (F) CTNS-1::mCherry in cell body and process of PLM neurons in WT, syd-2 and apb-3; syd-2 mutants that express the various SYD-2 constructs. Red arrows point to CTNS-1::mCherry signal in the PLM neuronal process. Scale bar: 10 μm. (G) Penetrance for the number of animals in which CTNS-1 is seen beyond 50 μm of the PLM neuronal process away from the cell body in WT, syd-2 and apb-3; syd-2 mutants that express the various SYD-2 constructs. (TIF) [file pgen.1011253.s005.tif]

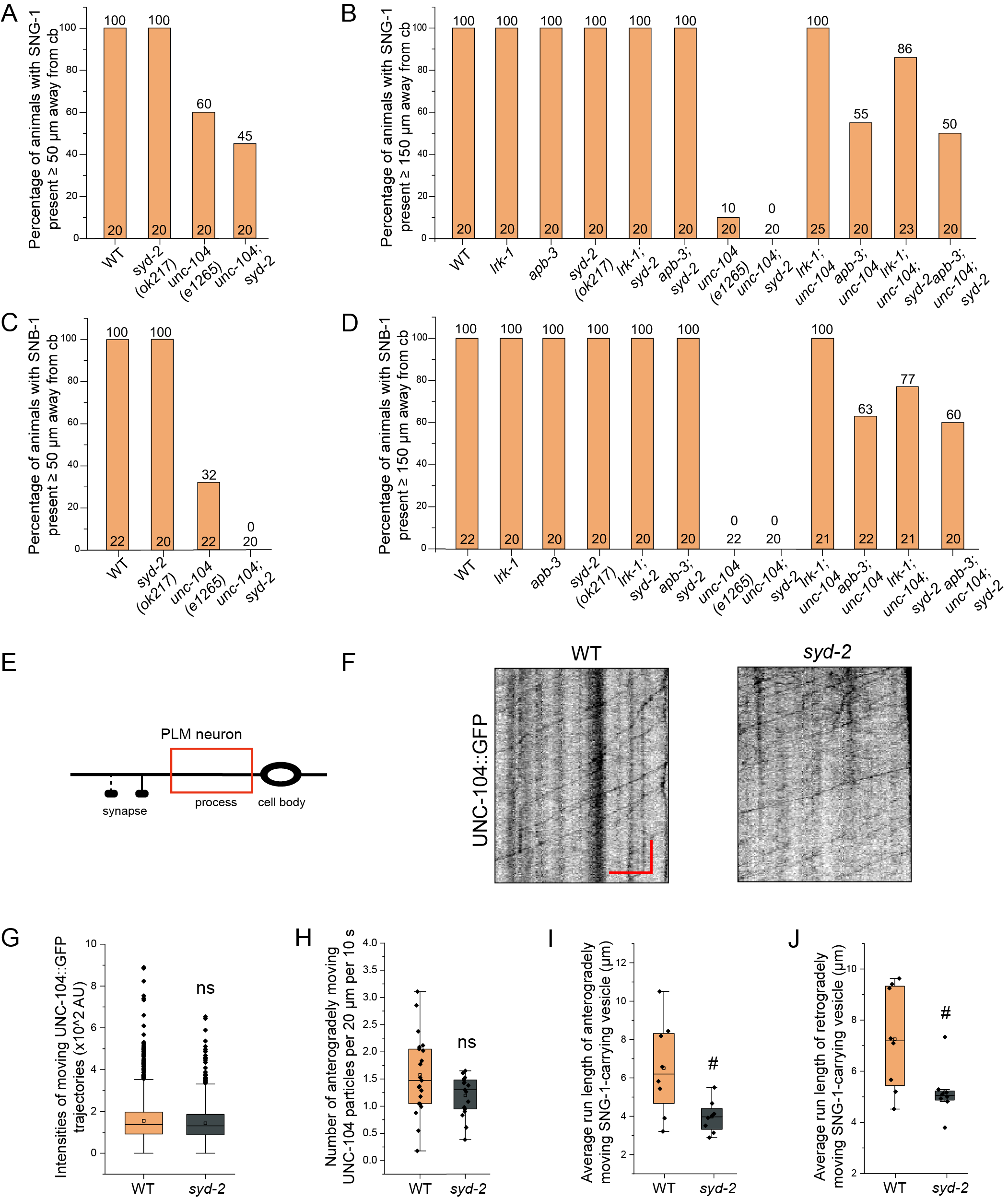

Supplement: S6 Fig — (A) Penetrance for the number of animals in which SNG-1 is seen beyond 50 μm of the PLM neuronal process away from the cell body in unc-104(e1265), syd-2(ok217) and unc-104; syd-2 double mutants. Numbers inside the bars indicate the number of animals per genotype. Numbers above the bars indicate the penetrance values. For very short bar graphs, the lower number indicates the number of animals for that genotype while the number above indicates the penetrance value. (B) Penetrance for the number of animals in which SNG-1 is seen beyond 150 μm of the PLM neuronal process away from the cell body in unc-104(e1265), syd-2(ok217) alone and in various mutant combinations with lrk-1(km17) and apb-3(ok429). Numbers inside the bars indicate the number of animals per genotype. Numbers above the bars indicate the penetrance values. For very short bar graphs, the lower number indicates the number of animals for that genotype while the number above indicates the penetrance value. (C) Penetrance for the number of animals in which SNB-1 is seen beyond 50 μm of the PLM neuronal process away from the cell body in unc-104(e1265), syd-2(ok217) and unc-104; syd-2 double mutants. Numbers inside the bars indicate the number of animals per genotype. Numbers above the bars indicate the penetrance values. For very short bar graphs, the lower number indicates the number of animals for that genotype while the number above indicates the penetrance value. (D) Penetrance for the number of animals in which SNB-1 is seen beyond 150 μm of the PLM neuronal process away from the cell body in unc-104(e1265), syd-2(ok217) alone and in various mutant combinations with lrk-1(km17) and apb-3(ok429). Numbers inside the bars indicate the number of animals per genotype. Numbers above the bars indicate the penetrance values. For very short bar graphs, the lower number indicates the number of animals for that genotype while the number above indicates the penetrance value. (E) Schematic of PLM neuron. Red box hig [file pgen.1011253.s006.tif]

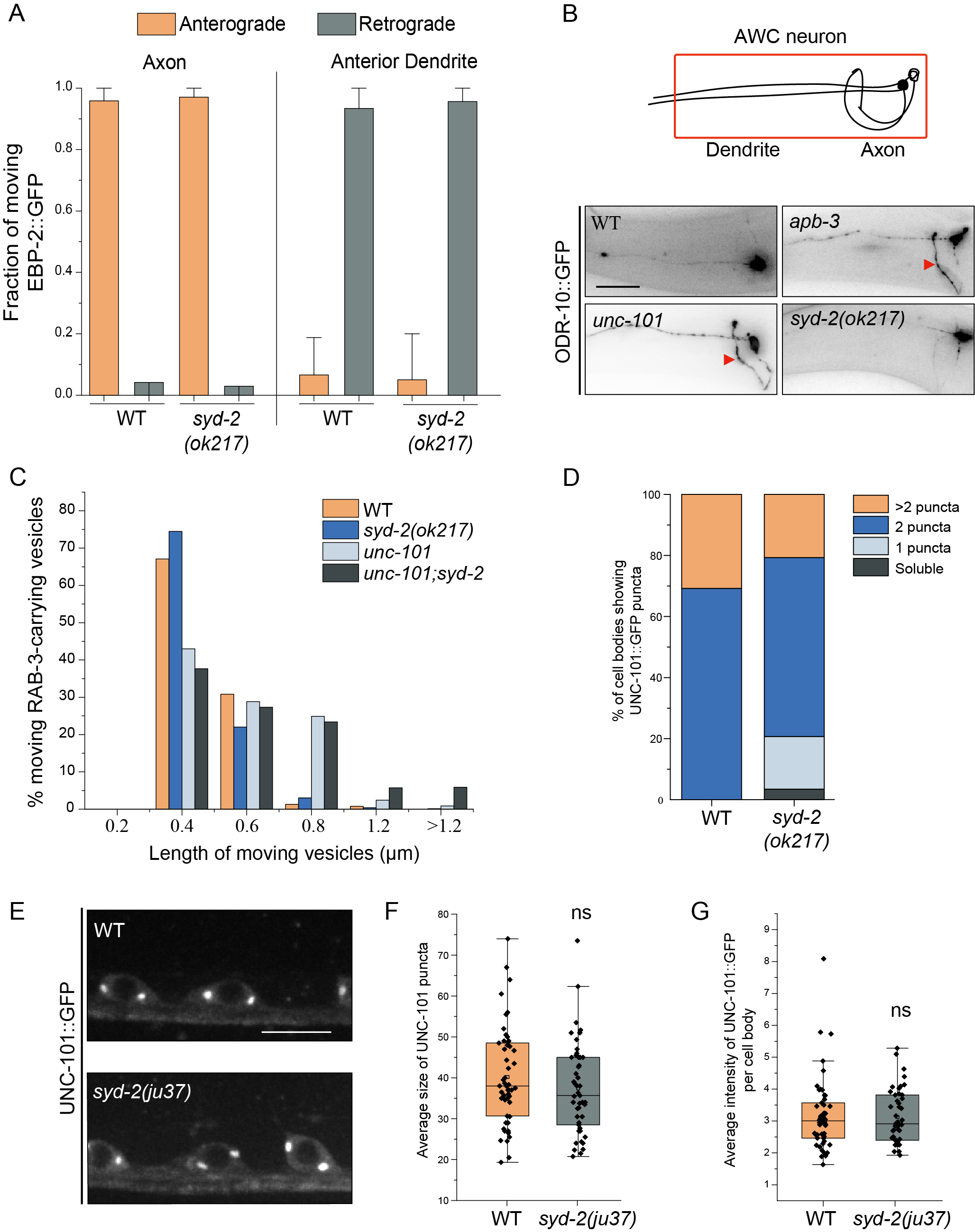

Supplement: S7 Fig — (A) Quantitation of fraction of EBP-2::GFP comets moving in either anterograde or retrograde directions in both the axon and the anterior dendrite of WT and syd-2(ok217); Number of animals (N) > 8 for each genotype; Number of comets analyzed (n) > 150. (B) Schematic of the AWC neuron with a red box highlighting the region of imaging. ODR-1::GFP in the dendrite and axon of the AWC neuron. Red arrow points to the ODR-1::GFP signal in the AWC axon in syd-2(ok217), apb-3(ok429), and unc-101(m1). Scale bar: 20 μm. (C) Quantitation of sizes of moving RAB-3 containing SVp carriers in WT, syd-2(ok217), unc-101(m1), and unc-101; syd-2. The x-axis depicts the length (in μm) of moving RAB-3 carrying SVp carriers. The y-axis depicts the percentage of moving RAB-3 carrying SVp carriers of various lengths. Number of animals (N) ≥ 9 per genotype; Number of vesicles (n) > 400. (D) Quantitation of the number of UNC-101::GFP puncta per cell body in WT and syd-2(ok217). P-value > 0.05 (Mann–Whitney Test); N > 5 animals; n > 25 cell bodies. (E) Images show UNC-101::GFP puncta in the cell bodies of the ventral nerve cord neurons in WT and syd-2(ju37). Scale bar: 10 μm. (F) Quantitation of the average size of UNC-101::GFP puncta per cell body in WT and syd-2(ju37). P-value > 0.05 (Mann–Whitney Test); ns: not significant; N > 5 animals; n > 25 cell bodies. (G) Quantitation of intensity of UNC-101::GFP puncta in the cell bodies of the ventral nerve cord in WT and syd-2(ju37). The ratio of the intensity of UNC-101::GFP puncta to cytosolic intensity in the cell body is plotted. P-value > 0.05 (Mann–Whitney test); ns: not significant; N > 5 animals; n > 10 cell bodies. (TIF) [file pgen.1011253.s007.tif]
